# The anterior and medial thalamic nuclei and the human limbic system: tracing the structural connectivity using diffusion-weighted imaging

**DOI:** 10.1038/s41598-020-67770-4

**Published:** 2020-07-02

**Authors:** Wolfgang Grodd, Vinod Jangir Kumar, Almut Schüz, Tobias Lindig, Klaus Scheffler

**Affiliations:** 1 Department of Magnetic Resonance, Max Planck Institute for Biological Cybernetics, Max Planck Ring 11, 72076 Tübingen, Germany; 2 Department of Neuroradiology, University Clinic Tübingen, Tübingen, Germany; 3 Department of Biomedical Magnetic Resonance, University Clinic Tübingen, Tübingen, Germany

**Keywords:** Nervous system, Brain

## Abstract

The limbic system is a phylogenetically old, behaviorally defined system that serves as a center for emotions. It controls the expression of anger, fear, and joy and also influences sexual behavior, vegetative functions, and memory. The system comprises a collection of tel-, di-, and mesencephalic structures whose components have evolved and increased over time. Previous animal research indicates that the anterior nuclear group of the thalamus (ANT), as well as the habenula (Hb) and the adjacent mediodorsal nucleus (MD) each play a vital role in the limbic circuitry. Accordingly, diffusion imaging data of 730 subjects obtained from the Human Connectome Project and the masks of six nuclei (anterodorsal, anteromedial, anteroventral, lateral dorsal, Hb, and MD) served as seed regions for a direct probabilistic tracking to the rest of the brain using diffusion-weighted imaging. The results revealed that the ANT nuclei are part of the limbic and the memory system as they mainly connect via the mammillary tract, mammillary body, anterior commissure, fornix, and retrosplenial cortices to the hippocampus, amygdala, medio-temporal, orbito-frontal and occipital cortices. Furthermore, the ANT nuclei showed connections to the mesencephalon and brainstem to varying extents, a pattern rarely described in experimental findings. The habenula—usually defined as part of the epithalamus—was closely connected to the tectum opticum and seems to serve as a neuroanatomical hub between the visual and the limbic system, brainstem, and cerebellum. Finally, in contrast to experimental findings with tracer studies, directly determined connections of MD were mainly confined to the brainstem, while indirect MD fibers form a broad pathway connecting the hippocampus and medio-temporal areas with the mediofrontal cortex.

## Introduction

**Figure 1:**
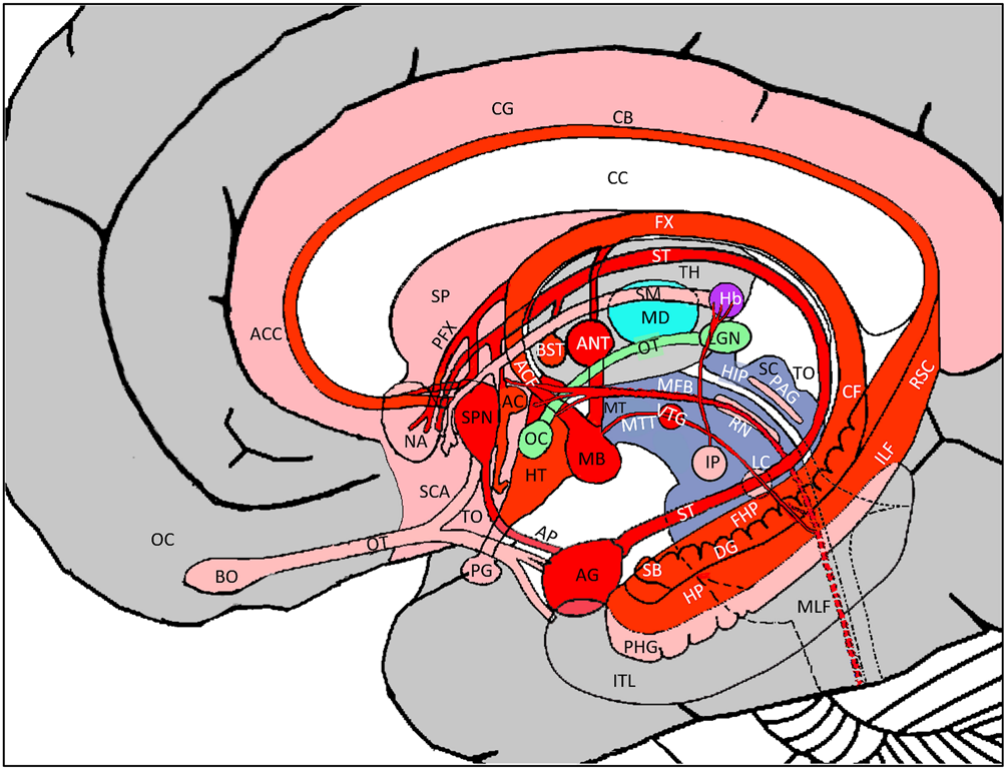
Proposed components of the limbic system. *Red*: Major structures of the classical circuit of Papez (937) defining a loop from the dentate gyrus (DG) of the hippocampus (HP) via the fornix (FX) to the mammillary body (MB) and via the mammillothalamic tract (MT) to the ANT. Light red: Extended concept of the limbic system as proposed by MacLean^108^ by incorporating both the Papez circuit and Yakovlev’s view^109^ of an amygdala–orbitofrontal network into a concept of the ‘visceral brain.’ *Light green*: Primary optic pathway. *Dark blue*: Mesencephalon. **Abbreviations:** *AC* anterior commissure, *ACC* anterior cingulate cortex, *ACF* anterior column of the fornix, *AG* amygdala, *AR* acustic radiation, *ANT* anterior nuclear group, *AP* ansa peduncularis, *ATP* anterior thalamic peduncle, *BST* bed nucleus of the stria terminals, *CF* crus fornicis, *CB* cingulum bundle, *CG* cingulate gyrus, *DG* dentate gyrus, *FX* fornix, *Hb* habenula, *HP* hippocampus, *HIP* habenulo-interpeduncular tract, *HT* hypothalamus, *IC* inferior colliculi, *ILF* inferior longitudinal fasciculus, *IP* nucleus interpenducularis, *ITL* inferior temporal lobe, *LGN* lateral geniculate nucleus, *MB* mammillary body, *MD* mediodorsal nucleus, *MFB* medial forebrain bundle, *MGN* medial geniculate nucleus, *MPC* medial prefrontal cortex, *MLF* medial longitudinal fasciculus, *MT* mammillothalamic tract, *MTT* mammillotegmental tract, *NA* nucleus accumbens, *NC* nucleus caudatus, *OC* optic chiasm, *OFC* orbito-frontal cortices, *OT* optic tract, *PC* parietal cortex, *PFX* Precommisural Fornix, *PG* postcentral gyrus, *PAG* periaqueductal grey, *PHG* parahippocampal gyrus, *RN* raphe nuclei, *RSC* retrosplenial cortex, *SB* subiculum, *SC* superior colliculi, *SCA* subcallosal area, *SI* substantia innominata, *SM* stria medullaris, *SP* septum, *SCP* superior cerebellar peduncle, *SCT* spinocerebellar tract, *SPN* septal nuclei, *ST* stria terminalis, *STP* superior thalamic peduncle, *TH* thalami nuclei, *TO* tectum opticum, *UF* uncinate fasciculus, *VC* visual cortices, *VTG* ventral tegmental nucleus of Gudden.

The limbic system is a phylogenetically old system that serves as a center for emotions, controlling our expressions of anger, fear, and joy and influencing sexual behavior, vegetative functions, and memory. It forms a double ring around the basal ganglia and the thalamus and encircles phylogenetically older parts of the cerebral cortex (allocortex), subcortical structures of the medial hemispheres and midbrain connections^1–5^. More specifically, it includes the hippocampus, fornix, corpus mamillare, cingulate bundle, amygdala, parahippocampal gyrus, septal areas, and the anterior nuclear group (ANT). However, as the limbic system is interconnected by the ascending and descending fibers with the medial mesencephalon, pons, and spinal cord, the habenula (Hb) and the dorso-medial thalamic nucleus (MD)—positioned between ANT and Hb—should also be considered as part of an extended limbic system^6–9^ and were therefore also included in the analysis (s. Fig. 1).

**Figure 2:**
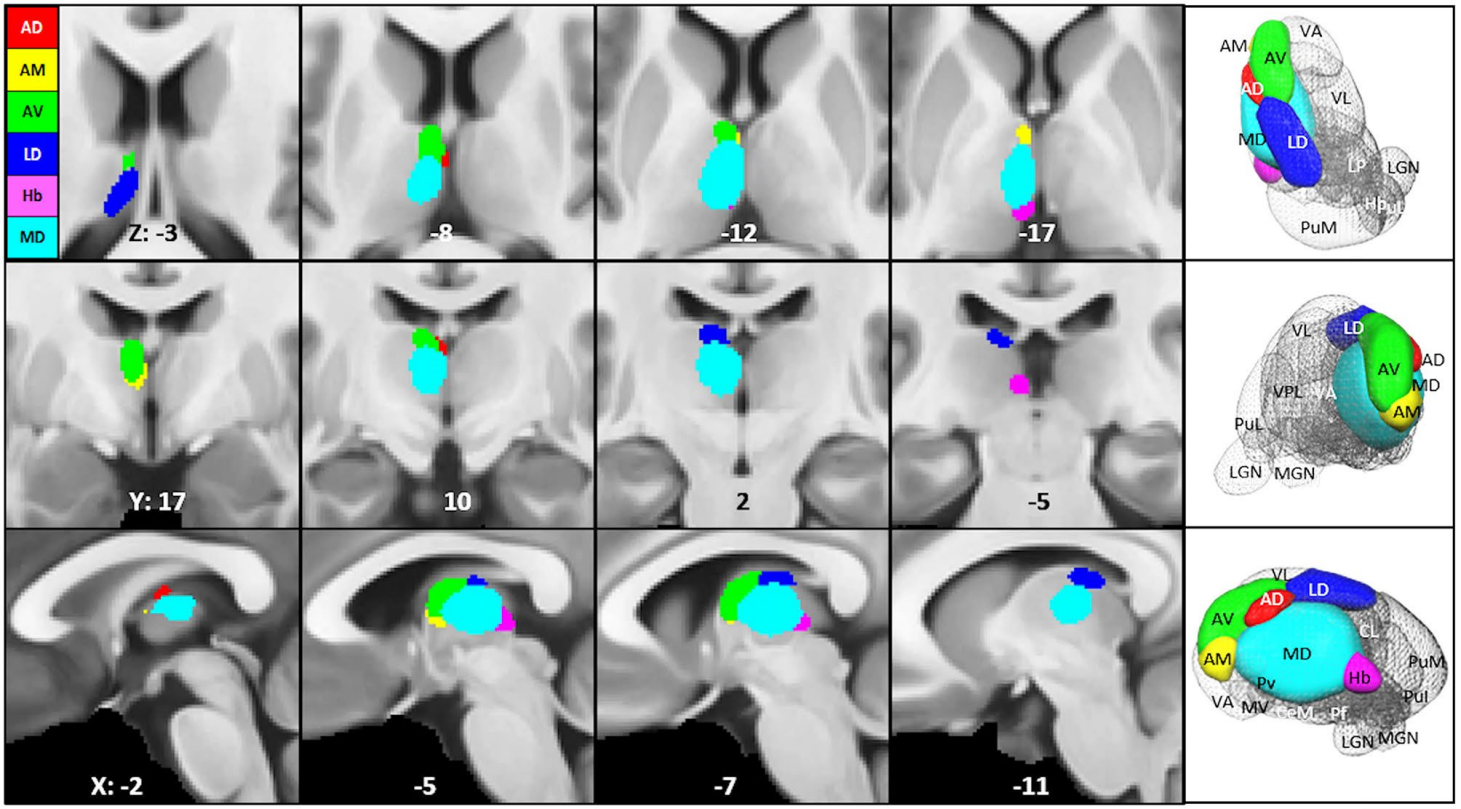
Location of the anterior nuclei, habenula, and mediodorsal nucleus of the thalamus according to Morel^32^ Left: Four axial, frontal, sagittal sections overlaid on the MNI brain template with corresponding X, Y, and Z coordinates. Right: Corresponding 3D rendered views of the selected nuclei overlaid on a thalamic mesh. *AD* anterior dorsal nucleus, *AM* anterior medial nucleus, *AV* anterior ventral nucleus, *LD* lateral dorsal nucleus, *Hb* habenula, *MD* mediodorsal nucleus.

The anterior nuclear group (ANT) located in the rostral one-third of the thalamus is considered to be a significant part of the limbic system and a component of the circuit of Papez^10^ as it has extensive hippocampal–anterior thalamic interconnections^11–13^. The ANT is easy to identify (s. Fig. 2), because it is separated from the rest of the dorsal thalamus by the internal medullary lamina (IML). It consists of four nuclei: the anterodorsal (AD), anteromedial (AM), and anteroventral (AV) nuclei within the IML and the adjacent lateral dorsal nucleus (LD). The LD nucleus borders the anterior group postero-laterally and occupies the most dorsal aspect of the thalamus; it is surrounded by a thin fiber capsule that facilitates its identification in all species^14^. LD belongs to the anterior nuclei group because it shares its topographic association and many of the limbic connections and has electrophysiological properties similar to those of the anterior dorsal thalamic nucleus.

The connectivity profile of the various nuclei of the thalamus, including the ANT, Hb, and MD, has been examined in numerous experimental and postmortem studies^15–17^. Mainly due to its dense connection with the hippocampus, the ANT has become a promising location for treatment with deep-brain stimulation (DBS) in patients with intractable seizures^18,19^. However, human in vivo examinations of the connectivity of these nuclei with the surrounding diencephalic nuclei and adjacent limbic and frontal cortices are rare. Due to the paucity of tightly defined and validated thalamic substructures that can serve as a starting point, most human studies have instead used a telencephalic approach to target thalamic connections. For example, diffusion-weighted imaging (DWI) was successful in identifying particular connections between the thalamus and pre-defined cortical areas^20–22^. Similarly, resting state fMRI (rsfMRI) has shown some significant functional relations of cortical areas with the thalamus^23–28^.

Due to the recent availability of in vivo data from large samples of the human brain, we decided to return to the direct approach by applying anatomically defined thalamic masks to a large sample of human in vivo data and tracking their connections to the adjacent diencephalic structures, cortical and mesencephalic areas. Accordingly, DWI data were taken from the Human Connectome Project^29^, and masks of the ANT, Hb, and MD nuclei were used as seed regions for a probabilistic tracking to the rest of the brain. This study presents the first attempt to examine and verify the structural connectivity between diverse components of the limbic system and selected thalamic nuclei in a whole brain approach in humans.

## Material and methods

### Data

Data used in the preparation of this work were obtained from the MGH-USC Human Connectome Project (HCP) database (https://ida.loni.usc.edu/login.jsp). The HCP project (Principal Investigators: Bruce Rosen, M.D., Ph.D., Martinos Center at Massachusetts General Hospital; Arthur W. Toga, Ph.D., University of Southern California, Van J. Weeden, MD, Martinos Center at Massachusetts General Hospital) is supported by the National Institute of Dental and Craniofacial Research (NIDCR), the National Institute of Mental Health (NIMH) and the National Institute of Neurological Disorders and Stroke (NINDS). Collectively, the HCP is the result of efforts of co-investigators from the University of Southern California, Martinos Center for Biomedical Imaging at Massachusetts General Hospital (MGH), Washington University, and the University of Minnesota^29^.

The DWI data were obtained from the 3 T Human Connectome Project. The data were selected from the HCP-900 sample, and only those volunteers were chosen, who went through the full MRI acquisition pipeline of 2 structural, 4 resting state, 7 tasks and 1 DWI session (s. https://protocols.humanconnectome.org/HCP/3T/imaging-protocols.html). This selection resulted in 730 volunteers, a total of 329 male and 401 female subjects in the age range of 22–37 (693 right-handed and 37 left-handed subjects).

### MR data specification

#### Structural imaging

T1w MPRAGE; TR 2,400 ms; TE 2.14 ms; TI 1,000 ms; Flip Angle 8°; field of view (FOV) 224 × 224; 256 slices, voxel size 0.7 mm isotropic; bandwidth 210 Hz/Px, IPAT 2; acquisition time 7:40 min.

#### Diffusion spectrum imaging (DSI)

DWI data were acquired by using a spin-echo EPI sequence, TR: 5,520 ms, TE: 89.5 ms, flip angle; 78°; voxel size: 1.25 mm isotropic, 111 slices, multiband factor: 3, echo spacing: 0.78 ms, b-values: 1,000, 2000, and 3,000 s/mm^2^. For details, see:^30,31^.

### Thalamus mask definition

As masks, we used the digital version of “Stereotactic Atlas of the Human Thalamus and Basal Ganglia”^32^, which contains a set of 29 thalamic nuclei assigned to four major groups (anterior, medial, posterior and lateral) (s. Table 1). The digital model of the 3-D anatomy of the thalamus was transformed into a thalamus connectivity-based probability atlas space^21,33^. The transformation has been described in detail in previous studies^13,34^.

**Table 1:** List of 29 thalamic nuclei according to [32] ordered in four nuclei group with their full name, abbreviations, and size in voxel.

Medial group	Abbreviations	Voxel	Lateral group	Abbreviations	Voxel
Mediodorsal nucleus	MD	246	Ventral posterior lateral nucleus	VPL	182
Magnocellular part	MDmc		Anterior part	VPLa	
Parvocellular part	MDpc		Posterior part	VPLp	
Medioventral nucleus	MV	24	Ventral posterior medial nucleus	VPM	48
Central lateral nucleus	CL	291	Ventral posterior inferior nucleus	VPI	48
Central medial nucleus	CeM	82	Ventral lateral nucleus	VL	368
Centre médian nucleus	CM	85	Ventral lateral anterior nucleus	VLa	
Paraventricular nucleus	Pv	14	Ventral lateral posterior nucleus	VLp	
Habenular nucleus	Hb	26	Dorsal part	VLpd	
Parafascicular nucleus	Pf	85	Ventral part	VLpv	
Subparafascicular nucleus	sPf	19	Ventral anterior nucleus	VA	194
**Posterior group**			Magnocellular part	VAmc	
Medial pulvinar	PuM	376	Parvocellular part	VApc	
Inferior pulvinar	PuI	35	Ventral medial nucleus	VM	72
Lateral pulvinar	PuL	110	**Anterior group**		
Anterior pulvinar	PuA	74	Anterior dorsal nucleus	AD	21
Lateral posterior nucleus	LP	72	Anterior medial nucleus	AM	33
Medial geniculate nucleus	MGN	74	Anterior ventral nucleus	AV	94
Suprageniculate nucleus	SG	40	Lateral dorsal nucleus	LD	64
Limitans nucleus	Li	72	Voxel	Sum	2,961
Posterior nucleus	Po	40		AVG	102
Lateral geniculate nucleus	LGN	72		MAX	376
				MIN	14

### Nuclei native space transformation

The 12 parameter affine transformation^35,36^ was computed for each volunteer’s non-diffusion image and the MNI-spaced standard brain. The resulting transformation matrix was applied to the left and right anterior thalamic nuclei to transform them into the native diffusion space. The nuclei transformation allowed further diffusion calculations into subject native space while maintaining high data quality and reducing registration interpolation errors^37^.

### Preprocessing and diffusion fit

The obtained HCP diffusion data were already reconstructed using a SENSE1 algorithm^38^. The DWI data was preprocessed within the HCP pipeline, including distortion correction^39–41^ and motion correction. The color-coded FA maps were computed for each subject using FDT DT-fit tools and then visually inspected for data quality.

### Multi-shell reconstruction

The data were reconstructed using a sun grid engine to drive multiple CPUs and a GeForce GTX TITAN (Cuda 7.5 and compute capability 3.5) GPU. To enable bedpost processing on the referred GPU^42^, we used a custom-compiled version of the diffusion reconstruction tool, bedpostx_gpu^43^.

In both the CPU and GPU version, similar parameter settings were deployed to run the whole-brain multi-shell reconstruction. In the multi-shell model^44^ the diffusion coefficient was modeled using a gamma distribution. The number of fibers per voxel was three. The rician noise replaced the default Gaussian noise. The sun-grid engine-enabled CPU and GPU processing was performed using bedpostx.

### Connectivity distribution

Probabilistic tractography was performed using FSL-*probtrackx*^42^*.* The probability algorithm can build up a histogram of the posterior distribution on the basis of streamline location or connectivity distribution^42^. The entire analysis was performed using a CPU version of probtrackx on the sun-grid engine and Nvidia Titan GPU using the GPU version of the code, i.e., probtrackx2_gpu^43^. The probtrackx parameters included the curvature threshold 80° (0.2), sample number 5000, step length 0.5, and a maximum number of steps of 2000. Each seed parcel’s tractogram was confined to the ipsilateral hemisphere. In the direct diffusion tractography, all streamlines passing through other thalamic nuclei were excluded to depict only directly routed connections to the ipsilateral cortex. The resulting tractograms were each normalized by dividing them by the *way total* and multiplying them by 100.

### Group fixed effect analysis

The B0 volume of each subject was registered to MNI 1 mm brain space. The resulting transformation matrix was then applied to the tractograms to align them in the MNI space. Group fixed effects analysis was performed across all subjects for each parcel. Line artifacts generated by the seed restriction mask were removed by manual masking. Finally, the fixed effect maps were thresholded at a level of 0.25 and visualized in FSLeyes. The rendered visualization of the group connectivity distribution was achieved using Freeview (with minimal threshold 0.1)^45^.

### Anatomical Atlas label assignments

The threshold group fixed effect maps (≥ 0.25) were investigated for their specific cortical, sub-cortical and cerebellar projected assignments. Labeling (≥ 0.2 atlas thresholds) was performed using the Harvard–Oxford Cortical and Subcortical Structural Atlas^46–49^ as well as the Jülich Histological Atlas^50–52^. The assignments were computed separately for left and right group maps.

## Results

### Anterodorsal nucleus AD

**Figure 3:**
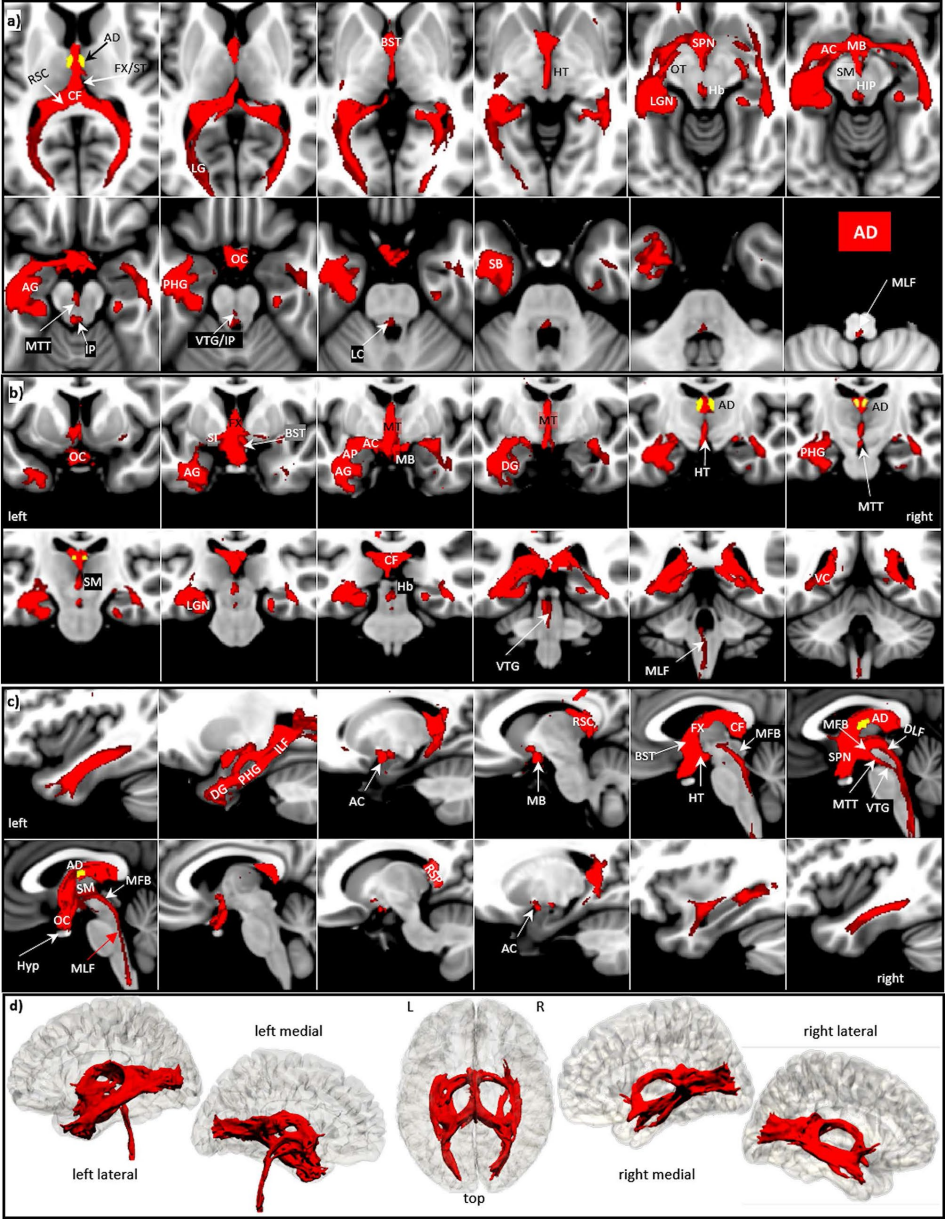
AD tracts in magnified **a** axial, **b** coronal axial, **c** sagittal views, and **d** 3D rendered views (threshold: 0.2–0.5) overlaid on the MNI brain. Note that the VTG and MTT traverse the mesencephalon and join the MLF at the floor of the fourth ventricle. **Abbreviations:** *AC* anterior commissure, *ACC* anterior cingulate cortex, *ACF* anterior column of the fornix, *AD* anterodorsal nucleus, *AG* amygdala, *AM* anteromedial nucleus, *ANT* anterior nuclear group, *AP* ansa peduncularis, *AR* acustic radiation, *ATP* anterior thalamic peduncle, *AV* anteroventral nucleus, *BST* bed nucleus of the stria terminals, *CB* cingulum bundle, *CF* crus fornicis, *CG* cingulate gyrus, *DG* dentate gyrus, *DLF* dorsal longitudinal fascicle, *DN* dentate nuclei, *DWI* diffusion-weighted imaging, *FX* fornix, *Hb* habenula, *HIP* habenulo-interpeduncular tract, *HP* hippocampus, *HT* hypothalamus, *IC* inferior colliculi, *ICP* inferior cerebellar peduncle, *ILF* inferior longitudinal fasciculus, *IML* internal medullary lamina, *IP* interpeduncular nucleus, *ITL* inferior temporal lobe, *ITP* inferior thalamic peduncle, *LC* locus coeruleus, *LD* lateral dorsal nucleus, *LG* lingual gyrus, *LGN* lateral geniculate nucleus, *LST* lateral spinothalamic tract, *MB* mammillary body, *MD* mediodorsal nucleus, *MFB* medial forebrain bundle, *MGN* medial geniculate nucleus, *MLF* medial longitudinal fasciculus, *MPC* medial prefrontal cortex, *MT* mammillothalamic tract, *MTT* mammillotegmental tract, *NA* nucleus accumbens, *NC* nucleus caudatus, *OC* optic chiasm, *OFC* orbito-frontal cortices, *OT* optic tract, *PAG* periaqueductal grey, *PHG* parahippocampal gyrus, *PC* parietal cortex, *PFX* Precommisural Fornix, *PG* postcentral gyrus, *PTP* posterior thalamic peduncle, *RN* raphe nuclei, *RSC* retrosplenial cortex, *rsfMRI* resting state fMRI, *SB* subiculum, *SC* superior colliculi, *SCA* subcallosal area, *SCP* superior cerebellar peduncle, *SCT* spinocerebellar tract, *SI* substantia innominata, *SM* stria medullaris, *SP* septum, *SPN* septal nuclei, *ST* stria terminalis, *STP* superior thalamic peduncle, *TH* thalami nuclei, *TO* tectum opticum, *UF* uncinate fasciculus, *VC* visual cortices, *VTG* ventral tegmental nucleus of Gudden.

The anterodorsal nucleus AD is the smallest nucleus of the ANT (21 voxels). It lies most medially and adjacent to the AV but is separated from AV, the stria medullaris thalami, and the ventricular surface of myelinated fibers and a glial lamella. Our results show that the major AD pathways reveal dominant projections to the left hemisphere. An overview of our results is given in axial, coronal, sagittal and 3D rendered views in Fig. 3 and described here in detail:

*The AD tracts connect:*

1. Rostro-caudally (a) along the mammillothalamic tract (MT) directly to the mammillary body (MB) and (b) indirectly via the anterior column of the fornix (FX) to the MB. The pre-commissural FX hereby comprises the septal region, including the bed nucleus of the stria terminals (BST), the pre-commissural septal nuclei (SPN), and the caudally adjacent hypothalamus (HT).
2. Via the anterior commissure (AC) bilaterally but left dominant to dentate gyrus (DG) and parahippocampal gyrus (PHG), hereby probably involving the superficial and laterobasal group of the left amygdala (AG) via the ansa peduncularis (AP).
3. A third major tract connects via the FX and the stria terminalis (ST) along the upper surface of the thalamus to the crus fornicis (CF) and retrosplenial cortex (RSC) and then (a) along the lingual gyrus (LG) to the visual cortices (VC), and (b) via the fimbria hippocampi and inferior longitudinal fascicle (ILF) anteriorly back along the para-hippocampal gyrus to the hippocampus and dentate gyrus.
4. Other significant tracts proceed bilaterally from the optic chiasm (OC) and the hypothalamus (HT) dorsally along the optic tract (OT) to the lateral geniculate nucleus (LGN) and then in a posterior direction along the posterior horn of the lateral ventricle to join the optic radiation and to end in the lingual gyrus (LG).
5. From the anterior commissure one track goes via the stria medullaris (SM) to the habenular nucleus (Hb) and from the habenula via the habenulo-interpeduncular tract (HIP) medioventrally to the aqueduct and the nucleus interpenducularis (IP), and via the caudally adjacent oculomotor nuclei to the medial mesencephalon, where it makes contact with the left locus coeruleus (LC) and then joins the medial longitudinal fasciculus (MLF).
6. Another medial tract connects the septal areas and the HT via the medial forebrain bundle (MFB) and the dorsal longitudinal fasciculus (DLF) with the medial mesencephalon, where it then joins the medial longitudinal fasciculus (MLF).
7. A final medial connection—the mamillotegmental tract (MTT)—connects the MB with the median mesencephalon, passing the interpeduncular nucleus (IP) and the adjacent raphe nuclei (RN), and then connecting via the ventral tegmental nucleus of Gudden (VTG) to the medial longitudinal fasciculus (MLF) at the floor of the fourth ventricle.
8. In the right hemisphere most tracts are much less pronounced, but in general take the same route anteriorly along the mammillary tract to the MB and via the anterior commissure to the hippocampus, where they are then more confined to medial aspects of the inferior temporal gyrus and project along the hippocampal gyrus without reaching its head and the amygdala. The posterior route along the body of the fornix and retrosplenial cortex into the lingual and occipital cortex is quite similar to the left hemisphere. However, on the right, the MTT tract is only recognizable at a lower threshold, and it remains unclear whether it also terminates in the VTG.

### Anteromedial nucleus AM

**Figure 4:**
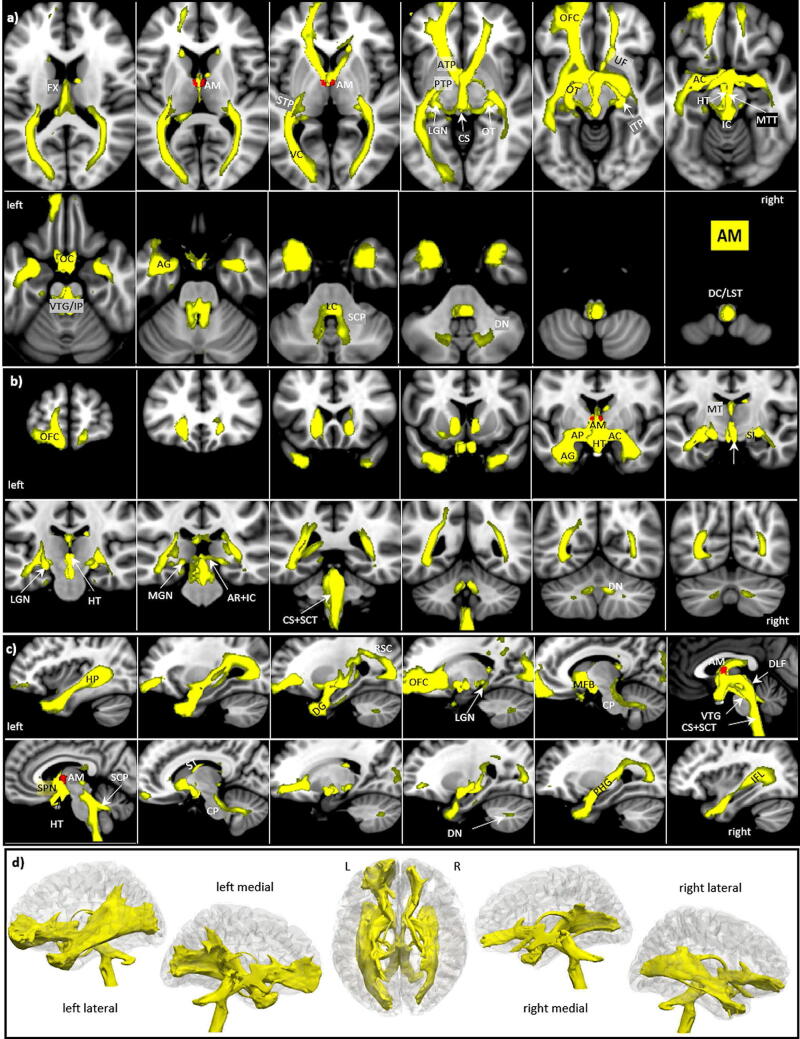
AM tracts in magnified **a** axial, **b** coronal axial, **c** sagittal views, and **d** 3D rendered views (threshold: 0.2–0.5) overlaid on the MNI brain MNI brain template. Note the right dominant stria terminals (black arrows in **d**) and the bilateral projection of the SCP into the cerebellum. **Abbreviations:** *AC* anterior commissure, *ACC* anterior cingulate cortex, *ACF* anterior column of the fornix, *AD* anterodorsal nucleus, *AG* amygdala, *AM* anteromedial nucleus, *ANT* anterior nuclear group, *AP* ansa peduncularis, *AR* acustic radiation, *ATP* anterior thalamic peduncle, *AV* anteroventral nucleus, *BST* bed nucleus of the stria terminals, *CB* cingulum bundle, *CF* crus fornicis, *CG* cingulate gyrus, *DG* dentate gyrus, *DLF* dorsal longitudinal fascicle, *DN* dentate nuclei, *DWI* diffusion-weighted imaging, *FX* fornix, *Hb* habenula, *HIP* habenulo-interpeduncular tract, *HP* hippocampus, *HT* hypothalamus, *IC* inferior colliculi, *ICP* inferior cerebellar peduncle, *ILF* inferior longitudinal fasciculus, *IML* internal medullary lamina, *IP* interpeduncular nucleus, *ITL* inferior temporal lobe, *ITP* inferior thalamic peduncle, *LC* locus coeruleus, *LD* lateral dorsal nucleus, *LG* lingual gyrus, *LGN* lateral geniculate nucleus, *LST* lateral spinothalamic tract, *MB* mammillary body, *MD* mediodorsal nucleus, *MFB* medial forebrain bundle, *MGN* medial geniculate nucleus, *MLF* medial longitudinal fasciculus, *MPC* medial prefrontal cortex, *MT* mammillothalamic tract, *MTT* mammillotegmental tract, *NA* nucleus accumbens, *NC* nucleus caudatus, *OC* optic chiasm, *OFC* orbito-frontal cortices, *OT* optic tract, *PAG* periaqueductal grey, *PHG* parahippocampal gyrus, *PC* parietal cortex, *PFX* Precommisural Fornix, *PG* postcentral gyrus, *PTP* posterior thalamic peduncle, *RN* raphe nuclei, *RSC* retrosplenial cortex, *rsfMRI* resting state fMRI, *SB* subiculum, *SC* superior colliculi, *SCA* subcallosal area, *SCP* superior cerebellar peduncle, *SCT* spinocerebellar tract, *SI* substantia innominata, *SM* stria medullaris, *SP* septum, *SPN* septal nuclei, *ST* stria terminalis, *STP* superior thalamic peduncle, *TH* thalami nuclei, *TO* tectum opticum, *UF* uncinate fasciculus, *VC* visual cortices, *VTG* ventral tegmental nucleus of Gudden.

The anteromedial nucleus AM is more ventrally and medially located than AD and appears as a ventromedial extension of AV with its lower part extending towards the third ventricle (s. Fig. 1). AM is also small in size (33 voxels). It is separated from AV by a thin fiber lamina and shows a somewhat higher density of myelinated fibers and cells. AM tracts cover similar diencephalic structures and areas as the AD. However, the tracts are much more extended in the frontal and occipital cortex and are also more prominent in the brainstem and the cerebellum. Nevertheless, again a left-sided dominance is maintained (s. Fig. 4).

*The AM tracts connect:*

1. Anteriorly they run much like the AD, but more prominently along the FX and MT to the MB, again including the hypothalamus (HT), all septal nuclei, and also the nucleus accumbens (NA). They then connect: (a) bilaterally via the anterior commissure to the hippocampus and via the ansa peduncularis (AP) to the entire amygdala (AG), which is also right dominant connected via the stria terminalis (ST), (b) posteriorly running via the retrosplenial cortex (RSC) and inferior longitudinal fascicle (ILF) back medio-frontally and then via the parahippocampal gyrus to the hippocampus, and (c) from the RSC to the visual cortices.
2. In the frontal direction, the tracts propagate from the MB and the septum bilaterally via the medial forebrain bundle (MFB) and the anterior thalamic peduncle (ATP), and from the temporal lobe via the uncinate fasciculus (UF) to the orbito-frontal cortices (OFC) until their frontal poles. For the most part they cover the orbito-frontal and ventromedial cortices (Brodmann area 10, 11 and 25) with a preponderance to the left. The MFB contains fibers running in both directions from the olfactory apparatus and the HT via the DLF to the brainstem nuclei, interchanging fibers with many nuclei along its way^53^.
3. Another prominent route runs posterior-medially adjacent to the posterior limb of the internal capsule along the superior thalamic peduncle (STP), which is divided into the inferior thalamic peduncle (ITP) and posterior thalamic peduncle (PTP) at the posterior circumference of the thalamus. The ITP connects the inferior colliculi (IC) with the medial geniculate nuclei (MGN) via the acoustic radiation (AR). Visual information from the optic chiasm (OC) and the optic tract (OT) reaches the LGN surrounding the mesencephalon and then via the PTP and optic radiation to the primary and secondary visual cortices (VC).
4. In the diencephalic center, a tract arises from the MB and connects directly to the medial mesencephalon via the hypothalamus (HT) and mammillo-tegmental tract (MTT). In doing so it passes the nucleus interpenducularis (IP) and the ventral tegmental nucleus of Gudden (VTG), ending at the floor of the fourth ventricle joining the medial longitudinal fasciculus (MLF).
5. Pronounced projections exist in the mesencephalon and brainstem which include all major nuclei and the ascending sensory dorsal column (DC) of the spinal cord and the lateral spinothalamic tracts (LST). One route starts from the MB and the adjacent hypothalamus (HT) and runs medially to the mesencephalon following two paths: (a) the mammillotegmental tract (MTT) propagating downwards to the dorsal mesencephalon and the VTG and further downwards most probably including the nuclei at the floor of the 4th ventricle like the Edinger Westphal, the oculomotor (III), and trochlearis (IV) nucleus, terminating in the MLF, and (b) the inferior and posterior thalamic peduncles to the brainstem, including the corticospinal tract (CS), and spinocerebellar tract (SCT), hereby entering the cerebellum via the superior cerebellar peduncle (SCP) to terminate in the dentate nuclei (DN).

### Anteroventral nucleus (AV)

**Figure 5:**
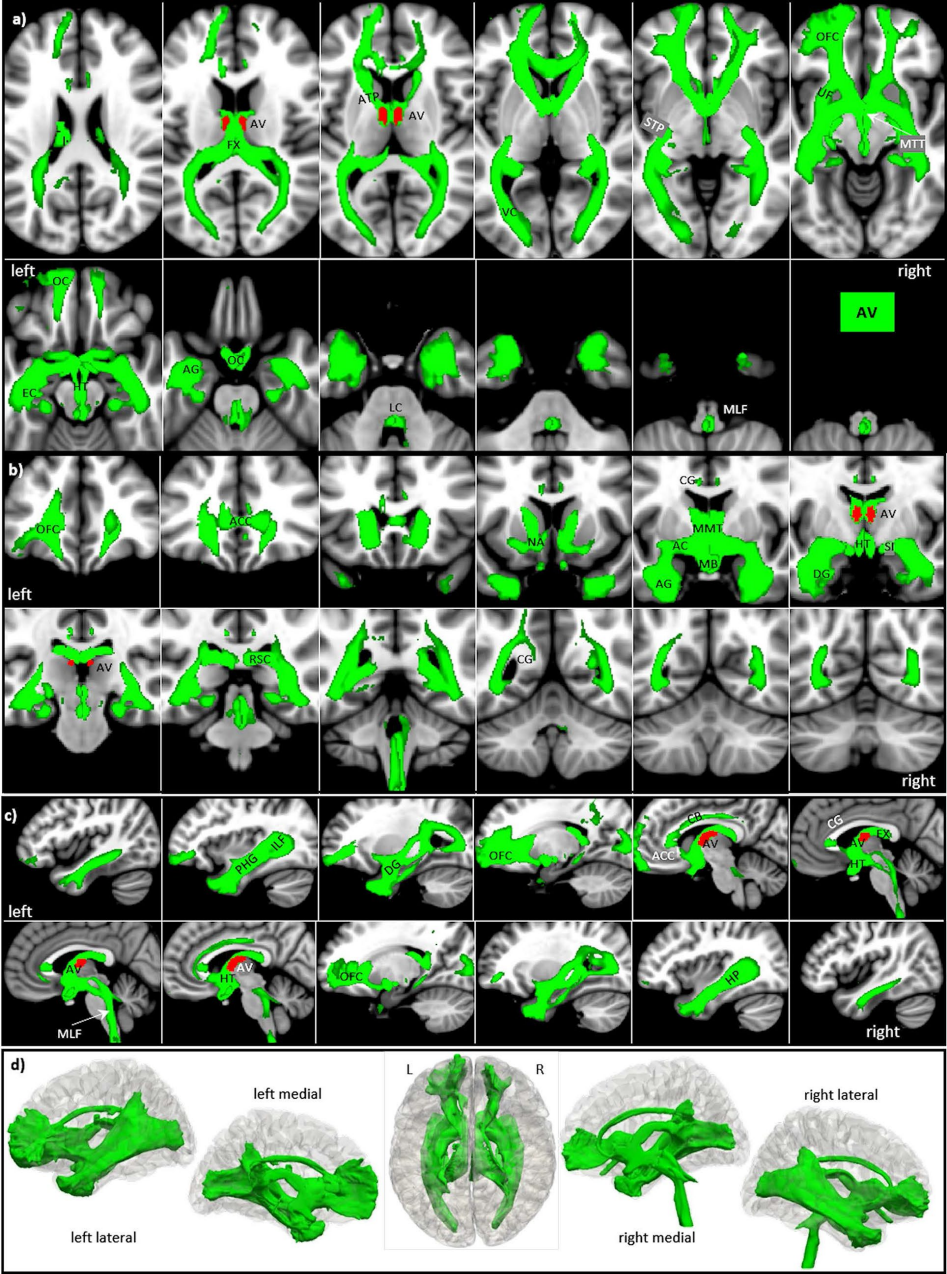
AV tracts in magnified **a** axial, **b** coronal axial, **c** sagittal views, and **d** 3D rendered views (threshold: 0.2–0.5) overlaid on the MNI brain template. Note the extended frontal projection including the anterior cingulate cortex and posterior projection via the cingulate bundle (black arrows in **d**). **Abbreviations:** *AC* anterior commissure, *ACC* anterior cingulate cortex, *ACF* anterior column of the fornix, *AD* anterodorsal nucleus, *AG* amygdala, *AM* anteromedial nucleus, *ANT* anterior nuclear group, *AP* ansa peduncularis, *AR* acustic radiation, *ATP* anterior thalamic peduncle, *AV* anteroventral nucleus, *BST* bed nucleus of the stria terminals, *CB* cingulum bundle, *CF* crus fornicis, *CG* cingulate gyrus, *DG* dentate gyrus, *DLF* dorsal longitudinal fascicle, *DN* dentate nuclei, *DWI* diffusion-weighted imaging, *FX* fornix, *Hb* habenula, *HIP* habenulo-interpeduncular tract, *HP* hippocampus, *HT* hypothalamus, *IC* inferior colliculi, *ICP* inferior cerebellar peduncle, *ILF* inferior longitudinal fasciculus, *IML* internal medullary lamina, *IP* interpeduncular nucleus, *ITL* inferior temporal lobe, *ITP* inferior thalamic peduncle, *LC* locus coeruleus, *LD* lateral dorsal nucleus, *LG* lingual gyrus, *LGN* lateral geniculate nucleus, *LST* lateral spinothalamic tract, *MB* mammillary body, *MD* mediodorsal nucleus, *MFB* medial forebrain bundle, *MGN* medial geniculate nucleus, *MLF* medial longitudinal fasciculus, *MPC* medial prefrontal cortex, *MT* mammillothalamic tract, *MTT* mammillotegmental tract, *NA* nucleus accumbens, *NC* nucleus caudatus, *OC* optic chiasm, *OFC* orbito-frontal cortices, *OT* optic tract, *PAG* periaqueductal grey, *PHG* parahippocampal gyrus, *PC* parietal cortex, *PFX* Precommisural Fornix, *PG* postcentral gyrus, *PTP* posterior thalamic peduncle, *RN* raphe nuclei, *RSC* retrosplenial cortex, *rsfMRI* resting state fMRI, *SB* subiculum, *SC* superior colliculi, *SCA* subcallosal area, *SCP* superior cerebellar peduncle, *SCT* spinocerebellar tract, *SI* substantia innominata, *SM* stria medullaris, *SP* septum, *SPN* septal nuclei, *ST* stria terminalis, *STP* superior thalamic peduncle, *TH* thalami nuclei, *TO* tectum opticum, *UF* uncinate fasciculus, *VC* visual cortices, *VTG* ventral tegmental nucleus of Gudden.

Despite its name, the anteroventral nucleus AV is more dorsally located than the AD and AM. AV is the largest ANT nucleus (94 voxels), and its cells are medium-sized, pale, and moderately densely packed. Anteriorly, it usually bulges into the lateral ventricle. Posteriorly, it is pushed deeper into the thalamus by the lateral dorsal nucleus LD. Compared to AM, the AV tracts show a similar but even a more extended pattern, again with a left-sided dominance (s. Fig. 5).

*The major AV tracts connect*:

1. Anteriorly like the AM along the FX and MT to the MB including the septal nuclei, the nucleus accumbens (NA), the preoptic area and the hypothalamus running via the anterior commissure to the hippocampus, including the whole amygdala (AG) and connecting more dorsally along the entire superior and medial temporal lobe via the fimbria hippocampi and inferior longitudinal fascicle.
2. Via the anterior thalamic radiation and uncinate fascicle (UF), connecting bilaterally to frontal areas, which now include almost the entire medial orbitofrontal, ventromedial prefrontal, dorsomedial prefrontal (Brodmann area 10, 11, 25 and 47), and the perigenual anterior cingulate cortex (ACC) (Brodmann area 12, 32).
3. A third major route connects orbitofrontal cortices and the septum dorsally via the fornix (FX) and cingulate bundle (CB) of the cingulate gyrus (CG) (a) with retrosplenial areas, terminating in the optic radiation and visual cortices, and (b) connecting via stria terminals (ST) with the amygdala^54^.
4. Another route runs along the posterior limb of the internal capsule and the superior thalamic radiation to (a) merge with other projections into the optic radiation and (b) return from retrosplenial areas to the posterior parahippocampal gyrus back to the hippocampus.
5. A final route connects from MB medially to VTG and MLF to the mesencephalon. It is mainly prominent on the right and runs medially along the floor of the 4th ventricle to the brainstem and spinal cord but without entering the cerebellum.

### Lateral dorsal nucleus (LD)

**Figure 6:**
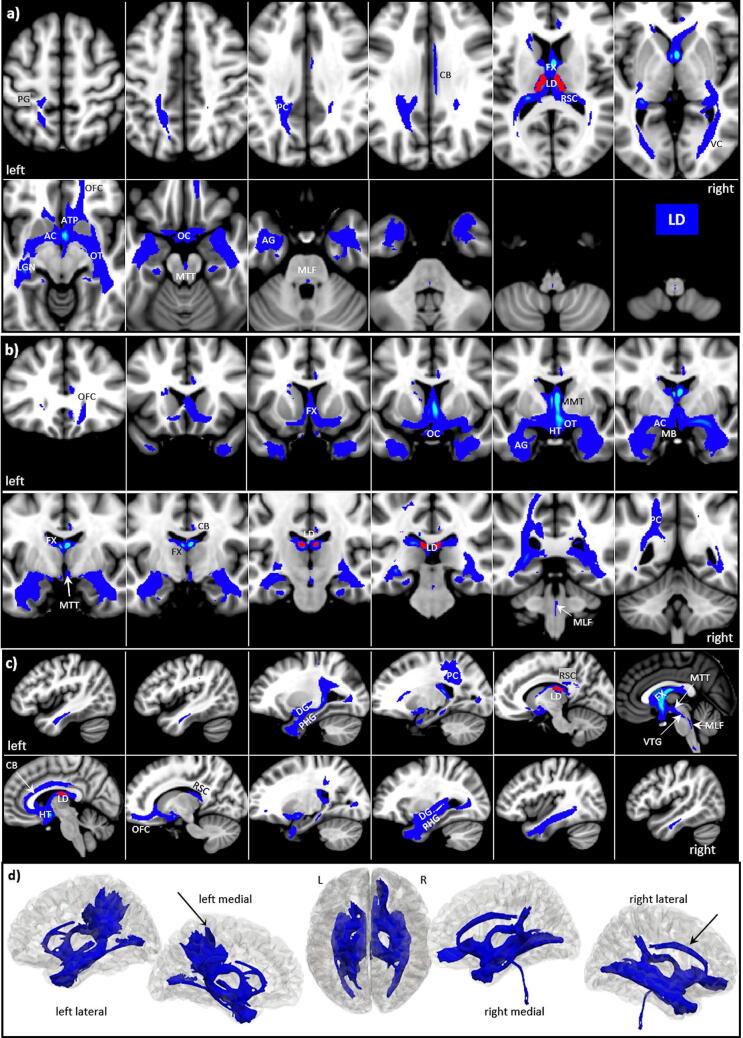
LD tracts in magnified **a** axial, **b** coronal axial, **c** sagittal views, and **d** 3D rendered views (threshold: 0.2–0.5) overlaid on the MNI brain. Note the exclusive projection along the cingulate bundle (black arrow in **d**) to the right orbitofrontal cortices, and the brainstem as well as to selective projection to the left postcentral gyrus. **Abbreviations:** *AC* anterior commissure, *ACC* anterior cingulate cortex, *ACF* anterior column of the fornix, *AD* anterodorsal nucleus, *AG* amygdala, *AM* anteromedial nucleus, *ANT* anterior nuclear group, *AP* ansa peduncularis, *AR* acustic radiation, *ATP* anterior thalamic peduncle, *AV* anteroventral nucleus, *BST* bed nucleus of the stria terminals, *CB* cingulum bundle, *CF* crus fornicis, *CG* cingulate gyrus, *DG* dentate gyrus, *DLF* dorsal longitudinal fascicle, *DN* dentate nuclei, *DWI* diffusion-weighted imaging, *FX* fornix, *Hb* habenula, *HIP* habenulo-interpeduncular tract, *HP* hippocampus, *HT* hypothalamus, *IC* inferior colliculi, *ICP* inferior cerebellar peduncle, *ILF* inferior longitudinal fasciculus, *IML* internal medullary lamina, *IP* interpeduncular nucleus, *ITL* inferior temporal lobe, *ITP* inferior thalamic peduncle, *LC* locus coeruleus, *LD* lateral dorsal nucleus, *LG* lingual gyrus, *LGN* lateral geniculate nucleus, *LST* lateral spinothalamic tract, *MB* mammillary body, *MD* mediodorsal nucleus, *MFB* medial forebrain bundle, *MGN* medial geniculate nucleus, *MLF* medial longitudinal fasciculus, *MPC* medial prefrontal cortex, *MT* mammillothalamic tract, *MTT* mammillotegmental tract, *NA* nucleus accumbens, *NC* nucleus caudatus, *OC* optic chiasm, *OFC* orbito-frontal cortices, *OT* optic tract, *PAG* periaqueductal grey, *PHG* parahippocampal gyrus, *PC* parietal cortex, *PFX* Precommisural Fornix, *PG* postcentral gyrus, *PTP* posterior thalamic peduncle, *RN* raphe nuclei, *RSC* retrosplenial cortex, *rsfMRI* resting state fMRI, *SB* subiculum, *SC* superior colliculi, *SCA* subcallosal area, *SCP* superior cerebellar peduncle, *SCT* spinocerebellar tract, *SI* substantia innominata, *SM* stria medullaris, *SP* septum, *SPN* septal nuclei, *ST* stria terminalis, *STP* superior thalamic peduncle, *TH* thalami nuclei, *TO* tectum opticum, *UF* uncinate fasciculus, *VC* visual cortices, *VTG* ventral tegmental nucleus of Gudden.

The lateral dorsal nucleus LD (64 voxels) can be macroscopically recognized as a slight elevation on the dorsal surface of the thalamus (between the fornix and tenia thalami). Like AV, LD is also separated from the rest of the thalamus through the superior wing of the IML. The LD tracts differ from the three other anterior nuclei by exhibiting a pronounced hemispheric difference, in which the orbitofrontal cortex is predominantly covered on the right, while a selective projection to the parietal postcentral cortex occurs only on the left (s. Fig. 6).

The major LD tracts connect:

1. Craniocaudally along the anterior column of the fornix and the MT to the MB, again including NA, the septal and hypothalamic nuclei, connecting bilaterally via the AC to the amygdala and hippocampus and then propagating dorsally along the dentate and parahippocampal gyrus and further to the retrosplenial cortex.
2. Anteriorly in a sparse connection restricted to the right anterior thalamic radiation to the gyrus rectus and the orbito-frontal cortex (OFC).
3. From the AC and the MTT one tract projects directly to the floor of the fourth ventricle, where it joins the nucleus of Gudden (VTG) and the medial longitudinal fasciculus (MLF).
4. A fourth route follows the fornix and the right cingulate bundle (CB) to the retrosplenial cortex, from which part of the tracts connect back to the hippocampus, linking a wide variety of cortical and subcortical sites to the hippocampus, see also^11^.
5. From the optic chiasm (OC) the optic tract (OT) surrounds the mesencephalon and joins the LGN, from which it then connects to the visual cortices (VC) via the posterior thalamic and optic radiation.
6. Finally, a selective tract occurs in the left hemisphere with a selective projection within the parietal cortex (PC) to the postcentral gyrus (PG) and temporoparietal junction.

### Habenula (Hb)

**Figure 7:**
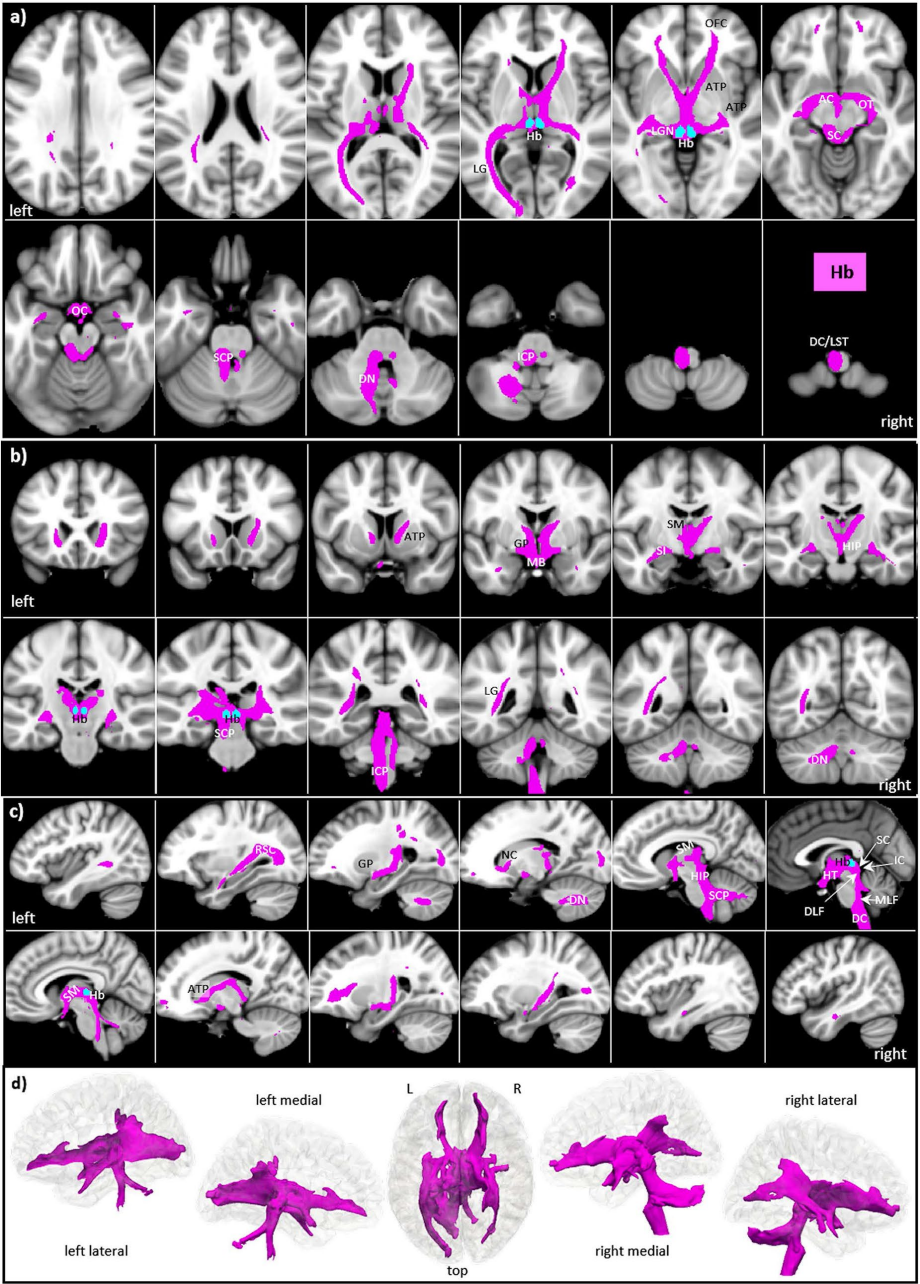
Hb tracts in magnified **a** axial, **b** coronal axial, **c** sagittal views, and **d** comparison of 3D rendered views of Hb (pink), and ANT nuclei (yellow) overlaid on the MNI brain template (threshold: 0.2–0.5). Note that the Hb tracts do not cover the anterior temporal lobes. **Abbreviations:** *AC* anterior commissure, *ACC* anterior cingulate cortex, *ACF* anterior column of the fornix, *AD* anterodorsal nucleus, *AG* amygdala, *AM* anteromedial nucleus, *ANT* anterior nuclear group, *AP* ansa peduncularis, *AR* acustic radiation, *ATP* anterior thalamic peduncle, *AV* anteroventral nucleus, *BST* bed nucleus of the stria terminals, *CB* cingulum bundle, *CF* crus fornicis, *CG* cingulate gyrus, *DG* dentate gyrus, *DLF* dorsal longitudinal fascicle, *DN* dentate nuclei, *DWI* diffusion-weighted imaging, *FX* fornix, *Hb* habenula, *HIP* habenulo-interpeduncular tract, *HP* hippocampus, *HT* hypothalamus, *IC* inferior colliculi, *ICP* inferior cerebellar peduncle, *ILF* inferior longitudinal fasciculus, *IML* internal medullary lamina, *IP* interpeduncular nucleus, *ITL* inferior temporal lobe, *ITP* inferior thalamic peduncle, *LC* locus coeruleus, *LD* lateral dorsal nucleus, *LG* lingual gyrus, *LGN* lateral geniculate nucleus, *LST* lateral spinothalamic tract, *MB* mammillary body, *MD* mediodorsal nucleus, *MFB* medial forebrain bundle, *MGN* medial geniculate nucleus, *MLF* medial longitudinal fasciculus, *MPC* medial prefrontal cortex, *MT* mammillothalamic tract, *MTT* mammillotegmental tract, *NA* nucleus accumbens, *NC* nucleus caudatus, *OC* optic chiasm, *OFC* orbito-frontal cortices, *OT* optic tract, *PAG* periaqueductal grey, *PHG* parahippocampal gyrus, *PC* parietal cortex, *PFX* Precommisural Fornix, *PG* postcentral gyrus, *PTP* posterior thalamic peduncle, *RN* raphe nuclei, *RSC* retrosplenial cortex, *rsfMRI* resting state fMRI, *SB* subiculum, *SC* superior colliculi, *SCA* subcallosal area, *SCP* superior cerebellar peduncle, *SCT* spinocerebellar tract, *SI* substantia innominata, *SM* stria medullaris, *SP* septum, *SPN* septal nuclei, *ST* stria terminalis, *STP* superior thalamic peduncle, *TH* thalami nuclei, *TO* tectum opticum, *UF* uncinate fasciculus, *VC* visual cortices, *VTG* ventral tegmental nucleus of Gudden.

In mammals, the Hb anatomically splits into two main subregions: the medial and lateral habenula, display distinct individual anatomical connectivity, see^55^. However, due to its small size, MRI usually cannot differentiate between the medial and lateral habenula^56^. Similarly, our Hb template was small (only 26 voxels) and contained both subdivisions. The significant tracts as visualized in our study are displayed in Fig. 7.

*The major Hb tracts connect*:

1. The major tracts run anteriorly bilaterally via the stria medullaris and fornix to the MB and the HT and via the anterior thalamic peduncle (ATP), probably including parts of the ventral pallidum (GP), to the central parts of the orbitofrontal cortices and the septum without involving the adjacent NA.
2. From MB only a few tracts connect via AC laterally and mediobasally. They involve the substantia innominata (SI) without reaching the medial and anterior parts of the temporal lobe.
3. In a posterior direction, the tracts encircle the inferior colliculi (IC) and superior colliculi (SC) of the lamina tecti and run via the optic tract posteriorly to the primary visual cortex.
4. From the MB there is also a habenulo-interpeduncular tract (HIP) to the Hb.
5. From the hypothalamus and septum, prominent connections run dorsocaudally but left dominant via the DLF to the mesencephalon and via superior cerebellar peduncle bilaterally to the cerebellum. Further downwards the tracts run left dominant along the dorsal columns (DC) and lateral spinothalamic tract (LST) of the spinal cord.

### Mediodorsal nucleus (MD)

**Figure 8:**
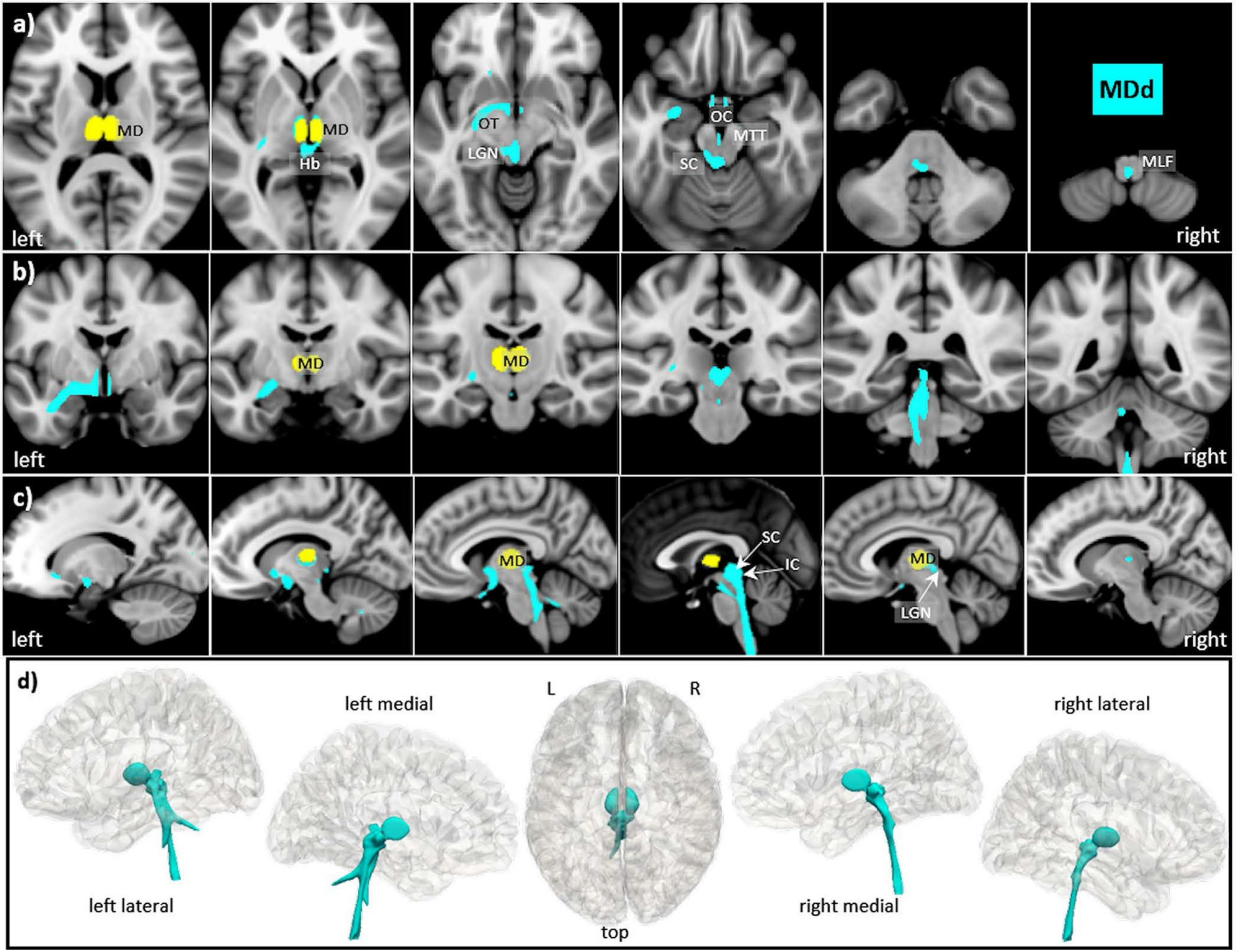
MD direct tracts (MDd) in magnified **a** axial, **b** coronal axial, **c** sagittal views, and **d** comparison of 3D rendered views of Hb (pink), and ANT nuclei (yellow) overlaid on the MNI brain template (threshold: 0.2–0.5). Note that all major tracts are confined to the mesencephalon and brainstem. **Abbreviations:** *AC* anterior commissure, *ACC* anterior cingulate cortex, *ACF* anterior column of the fornix, *AD* anterodorsal nucleus, *AG* amygdala, *AM* anteromedial nucleus, *ANT* anterior nuclear group, *AP* ansa peduncularis, *AR* acustic radiation, *ATP* anterior thalamic peduncle, *AV* anteroventral nucleus, *BST* bed nucleus of the stria terminals, *CB* cingulum bundle, *CF* crus fornicis, *CG* cingulate gyrus, *DG* dentate gyrus, *DLF* dorsal longitudinal fascicle, *DN* dentate nuclei, *DWI* diffusion-weighted imaging, *FX* fornix, *Hb* habenula, *HIP* habenulo-interpeduncular tract, *HP* hippocampus, *HT* hypothalamus, *IC* inferior colliculi, *ICP* inferior cerebellar peduncle, *ILF* inferior longitudinal fasciculus, *IML* internal medullary lamina, *IP* interpeduncular nucleus, *ITL* inferior temporal lobe, *ITP* inferior thalamic peduncle, *LC* locus coeruleus, *LD* lateral dorsal nucleus, *LG* lingual gyrus, *LGN* lateral geniculate nucleus, *LST* lateral spinothalamic tract, *MB* mammillary body, *MD* mediodorsal nucleus, *MFB* medial forebrain bundle, *MGN* medial geniculate nucleus, *MLF* medial longitudinal fasciculus, *MPC* medial prefrontal cortex, *MT* mammillothalamic tract, *MTT* mammillotegmental tract, *NA* nucleus accumbens, *NC* nucleus caudatus, *OC* optic chiasm, *OFC* orbito-frontal cortices, *OT* optic tract, *PAG* periaqueductal grey, *PHG* parahippocampal gyrus, *PC* parietal cortex, *PFX* Precommisural Fornix, *PG* postcentral gyrus, *PTP* posterior thalamic peduncle, *RN* raphe nuclei, *RSC* retrosplenial cortex, *rsfMRI* resting state fMRI, *SB* subiculum, *SC* superior colliculi, *SCA* subcallosal area, *SCP* superior cerebellar peduncle, *SCT* spinocerebellar tract, *SI* substantia innominata, *SM* stria medullaris, *SP* septum, *SPN* septal nuclei, *ST* stria terminalis, *STP* superior thalamic peduncle, *TH* thalami nuclei, *TO* tectum opticum, *UF* uncinate fasciculus, *VC* visual cortices, *VTG* ventral tegmental nucleus of Gudden.

**Figure 9:**
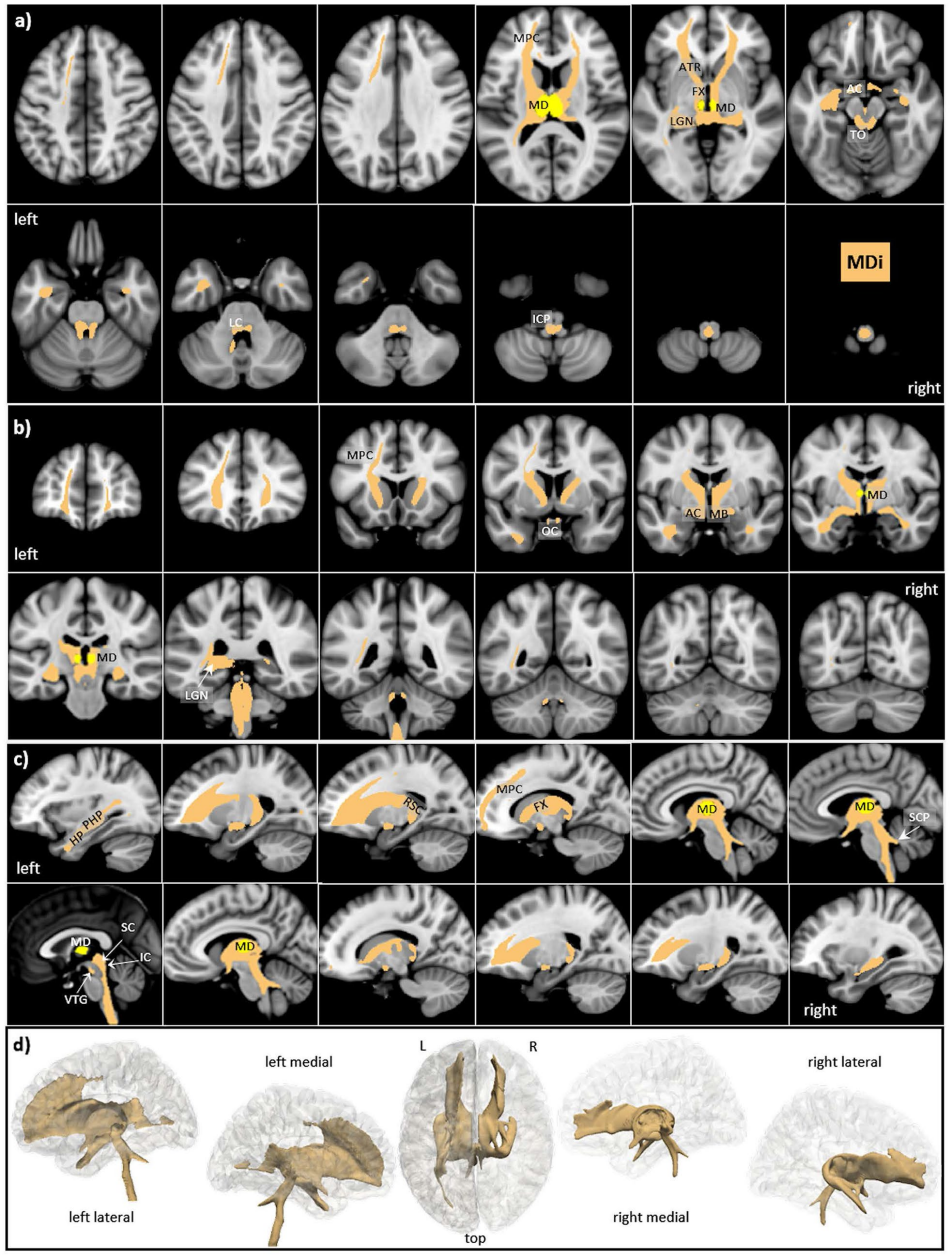
MD indirect tracts (MDi) in **a** axial, **b** coronal axial, **c** sagittal views, and **d** comparison of 3D rendered views of Hb (pink), and ANT nuclei (yellow) overlaid on the MNI brain template (threshold: 0.2–0.5). Note the almost exclusive and left dominant connection between the hippocampus via fornix and ANT nuclei to the mediofrontal cortices. **Abbreviations:** *AC* anterior commissure, *ACC* anterior cingulate cortex, *ACF* anterior column of the fornix, *AD* anterodorsal nucleus, *AG* amygdala, *AM* anteromedial nucleus, *ANT* anterior nuclear group, *AP* ansa peduncularis, *AR* acustic radiation, *ATP* anterior thalamic peduncle, *AV* anteroventral nucleus, *BST* bed nucleus of the stria terminals, *CB* cingulum bundle, *CF* crus fornicis, *CG* cingulate gyrus, *DG* dentate gyrus, *DLF* dorsal longitudinal fascicle, *DN* dentate nuclei, *DWI* diffusion-weighted imaging, *FX* fornix, *Hb* habenula, *HIP* habenulo-interpeduncular tract, *HP* hippocampus, *HT* hypothalamus, *IC* inferior colliculi, *ICP* inferior cerebellar peduncle, *ILF* inferior longitudinal fasciculus, *IML* internal medullary lamina, *IP* interpeduncular nucleus, *ITL* inferior temporal lobe, *ITP* inferior thalamic peduncle, *LC* locus coeruleus, *LD* lateral dorsal nucleus, *LG* lingual gyrus, *LGN* lateral geniculate nucleus, *LST* lateral spinothalamic tract, *MB* mammillary body, *MD* mediodorsal nucleus, *MFB* medial forebrain bundle, *MGN* medial geniculate nucleus, *MLF* medial longitudinal fasciculus, *MPC* medial prefrontal cortex, *MT* mammillothalamic tract, *MTT* mammillotegmental tract, *NA* nucleus accumbens, *NC* nucleus caudatus, *OC* optic chiasm, *OFC* orbito-frontal cortices, *OT* optic tract, *PAG* periaqueductal grey, *PHG* parahippocampal gyrus, *PC* parietal cortex, *PFX* Precommisural Fornix, *PG* postcentral gyrus, *PTP* posterior thalamic peduncle, *RN* raphe nuclei, *RSC* retrosplenial cortex, *rsfMRI* resting state fMRI, *SB* subiculum, *SC* superior colliculi, *SCA* subcallosal area, *SCP* superior cerebellar peduncle, *SCT* spinocerebellar tract, *SI* substantia innominata, *SM* stria medullaris, *SP* septum, *SPN* septal nuclei, *ST* stria terminalis, *STP* superior thalamic peduncle, *TH* thalami nuclei, *TO* tectum opticum, *UF* uncinate fasciculus, *VC* visual cortices, *VTG* ventral tegmental nucleus of Gudden.

The mediodorsal nucleus is a large nucleus which is easily identified by histology^57^ and in MRI^58,59^. It is located on the medial wall of the thalamus, abutting the third ventricle and possessing extensive connectivity to the prefrontal cortex, cingulate gyrus, and insula; see^57,60,61^. Based on cell morphology in rodents, MD can be divided into three different parts including medial MD, central MD, and lateral MD, with further subdivisions in primates; see^62^. In our thalamic template, MD—lacking further subdivision—was the largest nucleus (246 voxels). However, in contrast to the reported literature, our direct tracking revealed that all major connections are devoted to the mesencephalon and brainstem (s. Fig. 8). Therefore, we also analyzed all indirect tracts by tracing all MD connections which are routed via other thalamic nuclei (s. Fig. 9).

*Connections of the major direct MDd tracts*:

1. From the nucleus, the tracts run anteriorly via the MT and the MB to the optic chiasm (OC) and from MB left laterally via the AC to the anterior temporal pole without connections to DG and HP.
2. Posteriorly they run via the optic tract (OT) and LGN to the SC and IC of the lamina tecti, most probably including the Hb. They then propagate prominently but predominantly on the left to the dorsal mesencephalon and the brainstem.
3. Medially a subtle tract connects the hypothalamus via the MTT to the VTG, where it then joins the major mesencephalic pathways.

*Connections of the major indirect MDi tracts*:

1. Bilaterally in the frontal direction via the adjacent ANT and other nuclei (not shown) and bilaterally to the medio-frontal cortices (MFC) via the anterior thalamic radiation (ATR), with a clear preponderance to the left.
2. Via the pre-commissural fornix to MB and HT without involving the septum and adjacent nucleus accubens (NA) and then (a) bilaterally via AC but left dominant to the hippocampus and (b) similarly, left dominant in a posterior direction via the retrosplenial cortex, parahippocampal gyrus and the inferior longitudinal fascicle (ILF) back to the hippocampus.
3. Less prominent connections compared to the frontal ones run (a) posteriorly via the LGN and posterior thalamic radiation but do not reach the primary visual cortices and (b) medially via the stria medullaris to the Hb and tectum opticum (TO).
4. From the tectum and the medial mesencephalon, prominent tracts run bilaterally in a latero-dorsal direction via the superior cerebellar peduncle (SCP) into the cerebellum and further via the inferior cerebellar peduncle (ICP) to the spinal cord.

## Discussion

**Figure 10:**
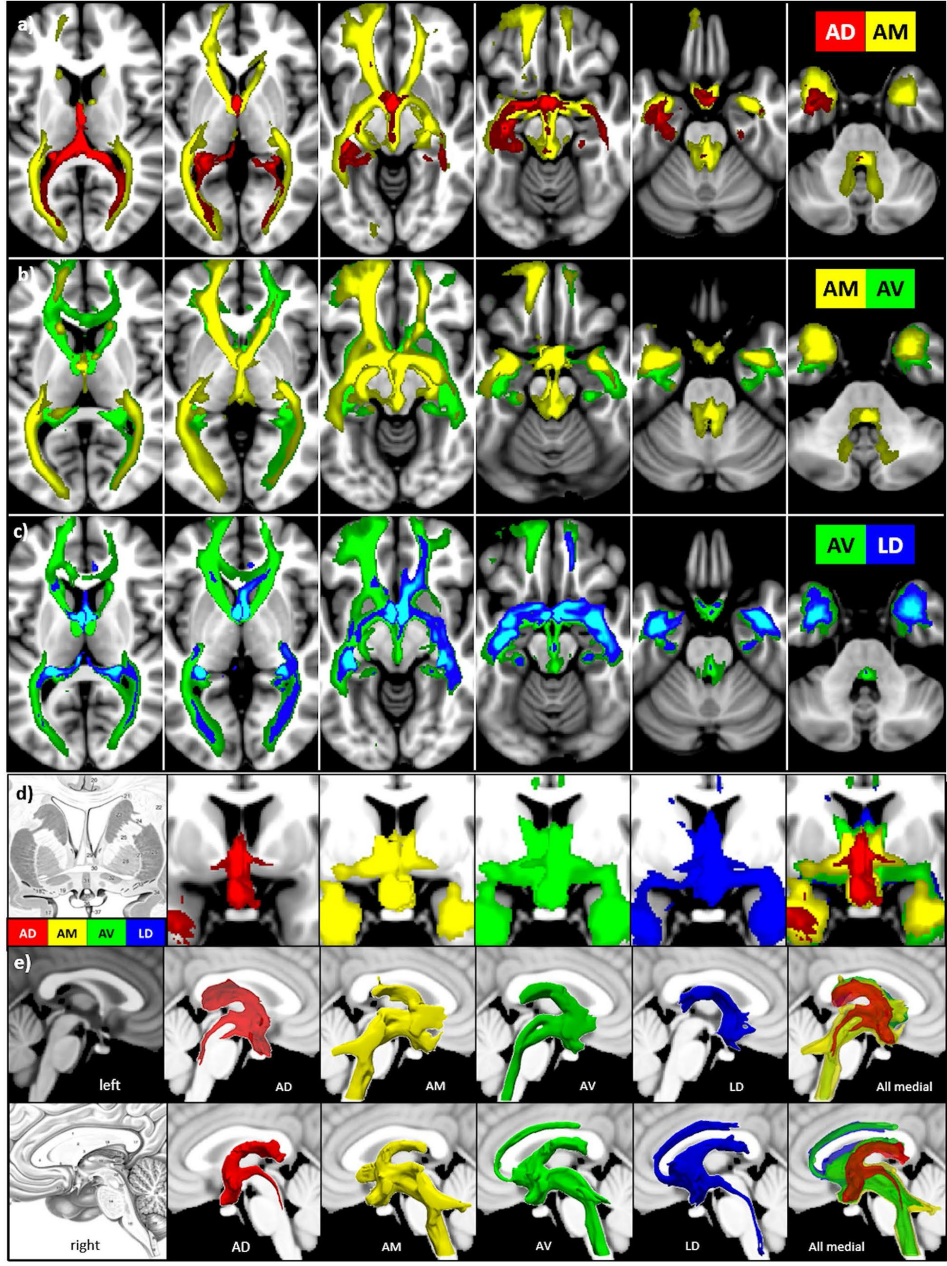
Comparison of the ANT nuclei. **a** AD versus AM, **b** AM versus AV, **c** AV versus LD, and **d**, **e** of all four nuclei in **d** coronal views at the level of the anterior commissure and **e** in midsagittal views for the left and right hemisphere (threshold: 0.15–0.3).

**Figure 11:**
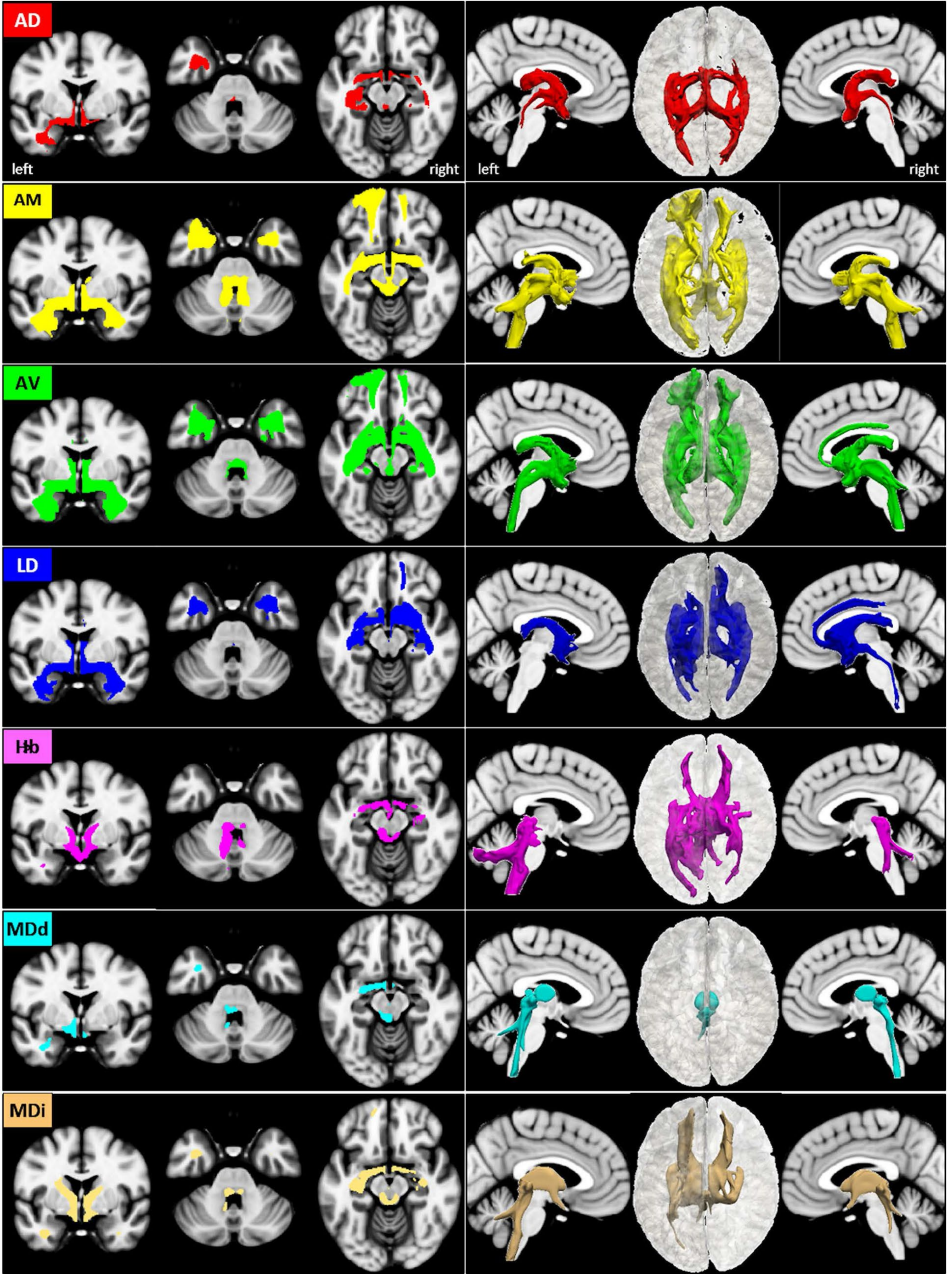
Comparison of all nuclei. Left: Corresponding coronal and axial views (threshold 0.2–0.5). Right: Rendered 3D views of the tracts in left and right parasagittal and top projections.

Although the mammillothalamic tract was already described in the eighteenth century by Vicq d’Azyr^63^, the thalamus was subsequently long neglected and regarded as simply being a part of the limbic system until Rose and Woosley^64^ correlated the “limbic cortex” with specific thalamic nuclei, based on experimental findings in rabbits. These results were later confirmed in numerous studies in various species^65^. Our study continues in this vein, aiming to determine how and to what extent selected *human* thalamic nuclei are *connected *in vivo to brain midline structures, which have been assigned to the limbic system^66^ since Broca (1878)^67^. An overview of the different connectivity patterns of the ANT nuclei is given in Fig. 10 and for all nuclei in Fig. 11. Moreover, Table 2 summarizes the major target structures for each nucleus according to the Juelich histology atlas, which is available electronically.

**Table 2:** Major connections of the anterior and medial thalamic nuclei in humans according to the Juelich histology atlas (https://fsl.fmrib.ox.ac.uk/fsl/fslwiki/Atlases/Juelich).

AD Left	AM Left	AV Left	LD Left	Hb Left	MD indirect Left
Amygdala_CM	Amygdala_CM	Amygdala_CM	Ant. intra-pariet. sulcus	Amygdala_CM	Amygdala_CM
Amygdala_LB	Amygdala_LB	Amygdala_LB	Amygdala_CM	Amygdala_LB	Amygdala_LB
Amygdala_SG	Amygdala_SG	Amygdala_SG	Amygdala_LB	Amygdala_SG	Amygdala_SG
HP cornu ammonis	HP cornu ammonis	HP cornu ammonis	Amygdala_SG	HP cornu ammonis	HP cornu ammonis
HP entorhinal cortex	HP entorhinal cortex	HP entorhinal cortex	HP cornu ammonis	HP dentate gyrus	HP dentate gyrus
HP dentate gyrus	HP dentate gyrus	HP dentate gyrus	HP entorhinal cortex	HP subiculum	HP subiculum
HP subiculum	HP subiculum	HP subiculum	HP dentate gyrus	Visual cortex V1 BA17	Acoustic radiation
Callosal body	Sup. parietal lobule	Visual cortex V1 BA17	HP subiculum	Visual cortex V2 BA18	Callosal body
Cingulum	Visual cortex V1 BA17	Visual cortex V3V	Prim. somatos. cortex	Visual cortex V3V	Corticospinal tract
Fornix	Visual cortex V2 BA18	Visual cortex V4	Sup. parietal lobule 5L	Visual cortex V4	Fornix
Lat. geniculate body	Visual cortex V3V	Callosal body	Sup. parietal lobule 7A	Acoustic radiation	Lat. geniculate body
Optic radiation	Visual cortex V4	Cingulum	Sup. parietal lobule 7P	Callosal body	Optic radiation
Uncinate fasc	Acoustic radiation	Fornix	Callosal body	Corticospinal tract	Sup. occ.-frontal fasc
Insula	Callosal body	Inf. occ.-frontal fasc	Corticospinal tract	Fornix	
	Corticospinal tract	Optic radiation	Fornix	Optic radiation	
	Fornix		Optic radiation	Sup. longitudinal fasc	
	Optic radiation		Sup. longitudinal fasc	Sup. occ.-frontal fasc	
	Uncinate fasc		Sup. occ.-frontal fasc	Insula	
	Cerebellum		Uncinate fasc		

AD Right	AM Right	AV Right	LD Right	Hb Right	MD indirect Right
HP cornu ammonis	Amygdala_CM	Amygdala_CM	Amygdala_CM	Amygdala_CM	Amygdala_CM
HP dentate gyrus	Amygdala_LB	Amygdala_LB	Amygdala_LB	Amygdala_LB	Amygdala_LB
HP subiculum	Amygdala_SG	Amygdala_SG	Amygdala_SG	Amygdala_SG	Amygdala_SG
Visual cortex V1 BA17	HP cornu ammonis	HP cornu ammonis	HP entorhinal cortex	HP cornu ammonis	HP cornu ammonis
Visual cortex V2 BA18	HP entorhinal cortex	HP entorhinal cortex	HP dentate gyrus	HP cornu ammonis	HP dentate gyrus
Callosal body	HP subiculum	HP dentate gyrus	HP subiculum	HP dentate gyrus	HP subiculum
Cingulum	Inf. parietal lobule PGp	HP subiculum	Prim. audit. cortex TE1	HP subiculum	Acoustic radiation
Fornix	Visual cortex V1 BA17	Visual cortex V1 BA17	Acoustic radiation	Visual cortex V1 BA17	Callosal body
Mammillary body	Acoustic radiation	Visual cortex V2 BA18	Callosal body	Acoustic radiation	Corticospinal tract
Optic radiation	Callosal body	Cingulum	Cingulum	Callosal body	Fornix
	Corticospinal tract	Fornix	Fornix	Corticospinal tract	Lat. geniculate body
	Fornix	Optic radiation	Optic radiation	Fornix	Mammillary body
	Optic radiation		Uncinate fasc	Lat. geniculate body	Optic radiation
	Cerebellum			Mammillary body	Sup. occ.-frontal fasc
				Optic radiation	
				Sup. occ.-frontal fasc	

### ANT

An evaluation of the connections reveals that all ANT nuclei are indeed associated with the limbic system, as they mainly include the hippocampal–diencephalic and parahippocampal–retrosplenial network dedicated to memory and spatial orientation (s. Fig. 10). In addition, AM, AV, and LD encircle the temporo-amygdala–orbitofrontal network involved in the integration of visceral sensation and emotion with semantic memory and behavior^82^. More specifically, all ANT nuclei encircle the MB, HT, and AC on their route to the hippocampus, but differ in their extent to the pre- and subcommissural septal areas as well as in their projections to the temporal pole and the adjacent amygdala. The AD in particular is mostly confined to the left anterior temporal lobe, and its connection via the MTT and VTG to the MLF and the brainstem is subtle. AM and AV both display broad connections to the dorsal brainstem but differ with respect to their connections with the cerebellum (only AM) and with the cingulum (only in right AV and LD); only AV includes the perigenual anterior cingulate cortex—a part of the default mode network (DMN). LD tracts—similar to AM—involve the right cingulum but offer—similar to AD—only a subtle connection via the right MTT to the brainstem. LD, however, differs from the other ANT nuclei in that it has a projection to the left parietal cortex, an area commonly not assigned to the limbic system. However, van Groen and Wyss^68^ reported after using anterograde tracer that in rats the lateroventral parts of LD project to the parietal cortex and that LD is attributed to spatial learning and memory^69^. This finding may correspond to the multimodal nature of the parietal cortex^70^.

### Hb

In contrast to the ANT, before MacLean^71^ the habenula was rarely recognized as part of the limbic system. Until today, the Hb is anatomically classified under the heading of the epithalamus^15–17^. The Hb is a highly conserved nucleus across vertebrates and has often been overlooked by neuroscientists. Its function was initially thought to be related to the regulation of the nearby pineal gland. Anatomically the Hb splits into two subregions in mammals: the medial and lateral habenula, which display distinct gene expression profiles and anatomical connectivity and hence are thought to subserve different functions. The medial habenula primarily receives input from the medial and lateral septal nuclei. Its output is almost entirely confined to the interpeduncular nucleus of the midbrain. On the other hand, the lateral habenula connects various structures, including the septum, hypothalamus, basal forebrain, globus pallidus, and prefrontal cortex, with the dopaminergic, serotonergic and noradrenergic systems^72^. Clinically Hb is of relevance to psychiatric disorders as a number of studies have associated it with dysregulated reward circuitry function, mood disorders, schizophrenia, and substance use disorder^9^.

We found that the Hb mainly connects via the stria medullaris and fornix with the hypothalamus and orbitofrontal cortices and posteriorly with the tectum and visual cortices. Caudally it is in contact with all major mesencephalic and brainstem nuclei and connects left dominantly with the cerebellum. Overall, these connections are consistent with experimental findings, in which most afferents to the habenular nuclei arrive via the stria medullaris. Afferents arising predominantly in limbic brain regions are directly or indirectly innervated by the lateral hypothalamic and lateral preoptic areas, basal forebrain structures, including the ventral pallidum, substantia innominata, and parts of the amygdala. The efferents mainly target the nuclei containing monoamine neurons in the brainstem like the dopaminergic ventral tegmental area (VTA) and substantia nigra pars compacta, serotonergic dorsal and median raphe, and cholinergic laterodorsal tegmentum^73–76^.

### MD

MD is one of the most frequently examined thalamic nuclei and extends (18–20 mm) as an ovoid structure from the level of the intrathalamic adhesion to the level of the habenular commissure. With its medial side bordering the third ventricle, MD is surrounded by the internal medullary lamina^57^. Therefore, the cortical pathways must cross other thalamic nuclei and cannot be visualized directly with DWI. Experimentally determined pathways using tracer substances reveal that MD has extensive connectivity to the prefrontal cortex, cingulate gyrus, and insula. Its frontal efferents are extensive^57,60,61,77^, so that Fuster^61^ defines the prefrontal cortex as cortical tissue having MD connectivity. MD plays a multifaceted role in higher cognitive functions in conjunction with the prefrontal cortex and other cortical and subcortical brain areas^78,79^. Specifically, it plays a role in recognition memory and familiarity based on inputs from the perirhinal cortex, and it is involved in the regulation of cortical networks, especially in cases in which the maintenance and temporal extension of persistent activity patterns in frontal lobe areas are required^80,81^. Based on cell morphology in rodents, MD can be divided into three different parts, the medial MD, central MD, and lateral MD, with further subdivisions in primates^62^.

As the direct MD tracking does not traverse other thalamic nuclei, we were only able to depict the reported extensive bilateral connections to the medio-frontal cortices (MFC) by adding all indirect connections.

### Connectivity differences between the nuclei

#### AD and AM

While the AD tracts are mainly confined to hippocampal–diencephalic, parahippocampal–retrosplenial, and the medial longitudinal fasciculus (MLF) and can be seen as being parts of a limbic core dedicated to memory and spatial orientation^82^, the AM connections extended farther into the orbito-frontal, temporal, and occipital regions. These AM frontal projections include the anterior temporal pole and the amygdala bilaterally, and the AM tracts project more broadly to the visual cortices and medial mesencephalon, the spinal cord, and the cerebellum. However, neither of these nuclei utilize the cingulate cortex to reach the retrosplenial cortex and to project back to the parahippocampal gyrus (s. Fig. 10).

#### AM and AV

In contrast to AD the AM and AV tracts show a very similar distribution pattern, with a slight dominance of the left hemisphere. However, AV extends bilaterally more orbito-frontally within the frontal lobe, especially to the upper part of superior frontal and towards the anterior cingulate gyrus, areas which are involved in the regulation of emotion, decision-making, self-control, and cognitive evaluation of morality^83,84^. In addition, the ventromedial prefrontal and perigenual anterior cingulate cortex are constituents of the default mode network^85,86^. Moreover, the medial connection now includes the cingulate bundle (s. Fig. 5). The connections to the mesencephalon and brainstem are quite similar. However, only AM connects via the superior cerebellar peduncle to the dentate nuclei and the adjacent cerebellum (s. Fig. 4).

#### AV versus LD

There are considerable differences between LD and the other three ANT nuclei. LD has prominent connections only to the right orbitofrontal cortex and a unique projection to the left parietal cortex. However, the involvement of the right cingulate bundle (CB) is quite similar to AV (s. Fig. 6). With respect to the brainstem, the connections are reduced to a fine component on the right, which passes the VTG and then joins the MLF.

#### ANT versus Hb and MD

In contrast to the ANT nuclei, the Hb tracts are sparse with respect to medio-temporal and occipital connections, but their brainstem and cerebellar connections are roughly as prominent as AM tracts (s. Fig. 11). The direct MD tracts are confined to the dorsal mesencephalon and spinal cord and lack significant cortical connections, in contrast to all other nuclei. However, allowing the MD tracts to reach the cortex via other nuclei reveals extensive bilateral connections to the medio-temporal lobe and the medio-frontal cortices (s. Fig. 9).

### Methodological limitations

This study had to deal with significant methodological problems, so the results are subject to major limitations.

First, the atlas of thalamic nuclei which we used is based on six series of maps derived from stacks of histologically processed brain sections by combining three different series of the right and left hemisphere to construct a unique three-dimensional surface rendered model of 29 major thalamic nuclei^32^. Therefore, the anatomical templates for each nucleus apply to both hemispheres, but cannot be seen as a representative sample for a larger population since they do not take the normal structural variation and hemispheric differentiation into account^87–89^. However, with respect to the connections to the frontal and temporal lobe, our tracking results are consistent with other human probabilistic tractography approaches^6,90^.

The human brain possesses a considerable variable organization within both hemispheres. Such as, the occipital cortex is more extended in the left hemisphere compared to the right and reversely the frontal cortex^91^. Such a slight asymmetric organization also appears for the thalamus^34,92^. For example, the left thalamus is more extended in the posterior direction in contrast to the right thalamus. To reflect this slight variability we now added bilateral images of the six examined nuclei for 2 subjects as an additional image in the supplement (S1) and the bilateral tacking results as additional images in the supplement (S2–S7). In a visual comparison, the maps matched closely with the group fixed effect maps.

Secondly, although DWI tractography is an important tool for determining structural pathways of the whole brain in vivo, uncertainty exists about the evaluation of spatial accuracy and anatomical assignment due to the inter-subject variability^93,94^. Seed-based probabilistic tractography can yield a mix of multi-brain area projections, in which a pathway connects from one node to the next and so on. Thus, the path can follow the probabilistic maxima defined by trajectories to the multiple brain areas. Intuitively, it is possible to estimate the direct path by adding the anatomically constrained node information into the seed-based tractography. However, we still lack a valid and detailed map of subcortical and brainstem structures and pathways, which are based on larger samples. Nevertheless, we hope that the obtained results can serve as roadmaps for a more detailed connectivity profile of the limbic system and the involved thalamic nuclei.

Thirdly, the anatomical assignment, especially of subcortical structures is limited as significant differences exist in the nomenclature and concepts for naming subcortical tracts and nuclei due to historical and experimental reasons^95,96^. Nonetheless, the described whole brain ANT connections extend existing experimental and anatomical findings on the limbic system, in particular concerning the brainstem. It seems that the anterior thalamus serves as a gating and control unit that transmits elementary functions associated with vision, hearing, motor control, sleep and wake cycles, alertness, and vegetative function from various brainstem centers to the diencephalon as well as to temporal and frontal cortices^11,12,97–100^.

### Interpretation

The interpretation of our results is imperfect in two ways. First, we had to link the connection between two different neuroscientific items: the thalamus—a circumscribed anatomical structure—and the limbic system—for the most part a behaviorally defined system whose components have evolved and increased over time^4,82^ and which is not universally accepted as a separate entity in the neurosciences^101–103^. Secondly, we had to compare our macroscopic whole brain results from a large sample of subjects with particular microscopic findings mainly determined in animals. The classical limbic circuit of Papez defines a loop from the hippocampal formation (dentate gyrus and subiculum) and parahippocampal gyrus via the post-commissural fornix (FX) to the mamillary body (MB) and then projects (a) via the mammillothalamic tract (MT) to the ANT^63^, (b) from ANT via the subcallosal and precallosal portions of the septum to septal and preoptic areas and via the cingulate bundle (CB) to the cingulate gyrus (CG), and (c) from the CG via the fimbria hippocampi and tractus perforans back to the hippocampus^5,12,104–106^. MacLean^107,108^ has widened this concept for a unitary model of the limbic system by incorporating both the Papez circuit and Yakovlev’s view^109^ of an amygdala–orbitofrontal network to develop a concept of the `visceral brain` considering that stimulation of the cingulate cortices can evoke autonomic changes that are linked to emotion (s. Fig. 2). As the ANT nuclei are considered as part of the limbic thalamus and a central component of the circuit of Papez^10^ with extensive direct and indirect hippocampal–anterior thalamic connections^11–13^, we were stimulated to analyze their connection profiles in a whole-brain approach. The inclusion of Hb and MD nuclei in our study is based on the fact that the Hb, due to its unique position, serves as a crossroad between the forebrain and midbrain regions^110–112^ and acts as a critical neuroanatomical hub that connects and regulates motivated behavior, affective states, cognition, and social behavior. Similarly, the MD serves as a primary cortical relay for the limbic system in offering major connections to the prefrontal cortex^113–115^.

## Conclusion

This study presents the first approach in humans to examine and verify the structural connectivity between diverse components of the limbic system and selected thalamic nuclei in a whole-brain approach. Despite methodological discrepancies between diffusion-guided fiber tracking and experimental connectivity studies using ante- and retrograde tracers in animals, we were able to confirm that the ANT, Hb, and MD nuclei connect—to different extents—to major limbic components and the mesencephalon and brainstem. While ANT nuclei broadly connect the hypothalamus, septal and prefrontal areas via fornix and cingulum with the retrosplenial area and the hippocampus, the Hb links the hypothalamus and orbitofrontal cortices via the stria medullaris and fornix to the tectum and visual cortices. Finally, only indirect MD tracts (i.e., with connectivity via other thalamic nuclei) show extensive bilateral connections to the medio-frontal cortex. The tracts of the six nuclei examined will be made available at https://github.com/vinkrishna/Limbic_Thalamus.

## Supplementary information

Supplementary file 1 (PDF 3425 kb)

## Data Availability

The study was performed in agreement with the WU-Minn HCP Consortium Open Access Data Use Terms of the Human connectome project. The study used datasets from the Human connectome project (HCP). We obtained HCP data use permission under open data use terms. Therefore, no further ethical approval was required. The HCP project (https://www.humanconnectomeproject.org/) is an open NIH initiative and got the required ethics approval for data acquisition and public distribution.
