# Supplementary material for: The anterior and medial thalamic nuclei and the human limbic system: tracing the structural connectivity using diffusion-weighted imaging
Source: Sci Rep. 2020 Jul 2;10:10957. doi: 10.1038/s41598-020-67770-4 (PMC7331724; doi:10.1038/s41598-020-67770-4)
Supplement: Supplementary file 1 — (PDF 3425 kb) [file 41598_2020_67770_MOESM1_ESM.pdf]

Supplement figures S1-S7 of AD, AM, AV, LD, Hb, and MD nuclei
from subject # 100206 subject # 100307

The Anterior and Medial Thalamic Nuclei and the Human Limbic System: Tracing the Structural Connectivity Using Diffusion-Weighted Imaging

Wolfgang Grodd[1*]

Vinod Jangir Kumar[1*],

Almut Schüz[1],

Tobias Lindig[1,2]

Klaus Scheffler[1,3],

[1]Max Planck Institute for Biological Cybernetics, Tübingen, Germany

[2]Department of Neuroradiology, University Clinic Tübingen, Germany

[3]Department of Biomedical Magnetic Resonance, University Clinic Tübingen, Germany

Draft to be submitted to Nature Scientific Reports
*these authors contributed equally
Corresponding author:
Prof. Dr. Wolfgang Grodd
Dept. of Magnetic Resonance
Max Planck Institute for Biological Cybernetics
Max Planck Ring 11
72076 Tübingen, Germany
Tel: +49 (0)7071 601-940
Fax: +49 (0)7071 601-702
Wolfgang.Grodd@tuebingen.mpg.de

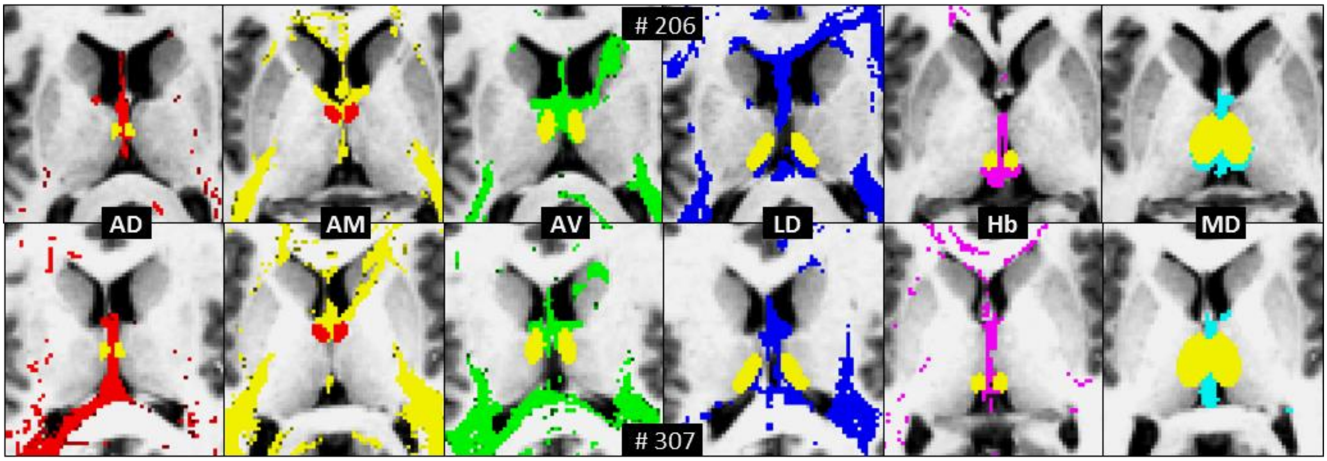

Suppl. Figure 1: Template for six thalamic nuclei. Bilateral projection of each nucleus on individual axial images of subject # 206 and # 307.

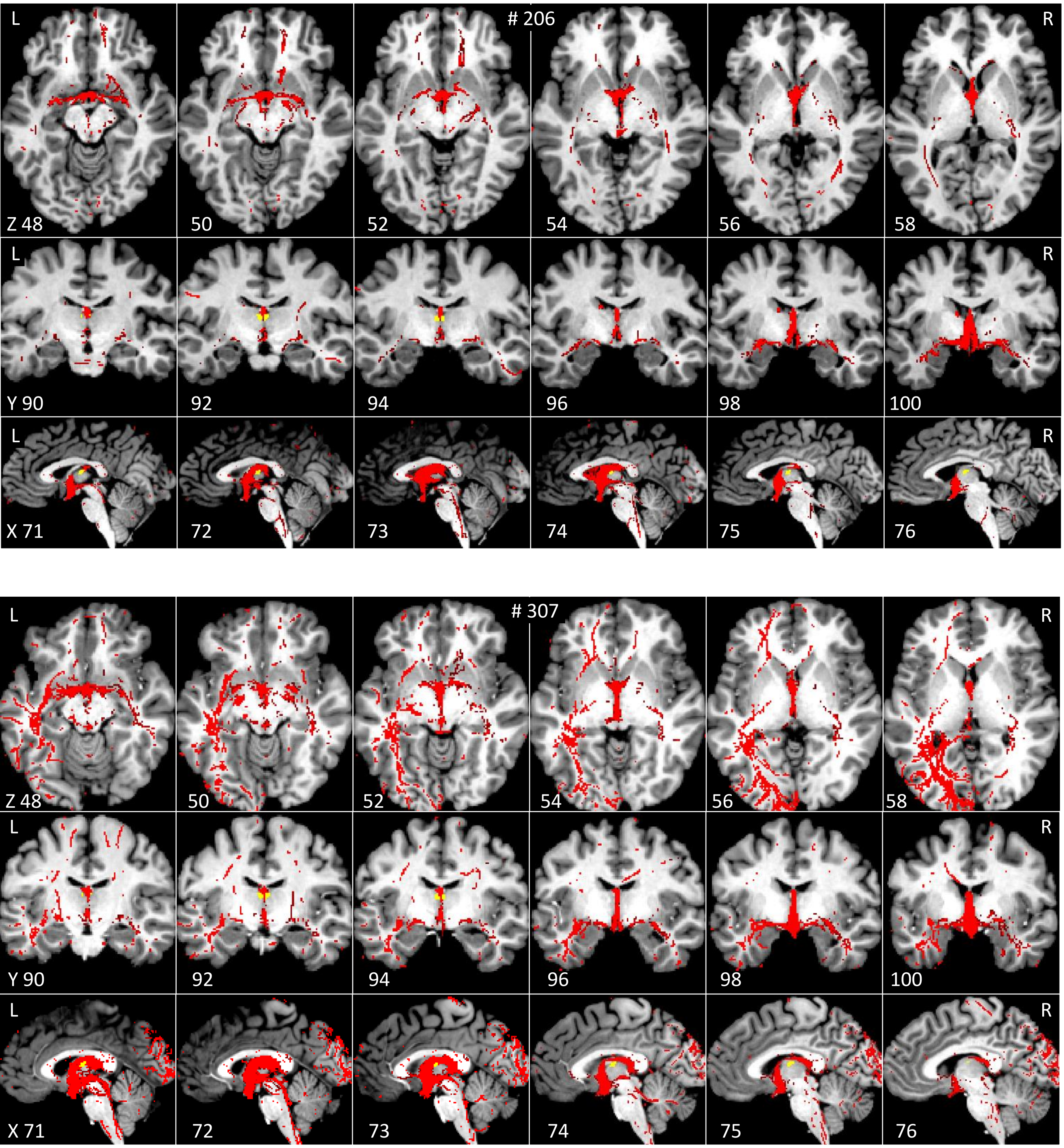

Suppl. Figure 2: Comparison of AD tracts of subject # 100206 and # 100307 in a) axial, b) coronal axial, c) sagittal views with the assignment of the slice positions in native diffusion space (T1 aligned to non diffusion image).

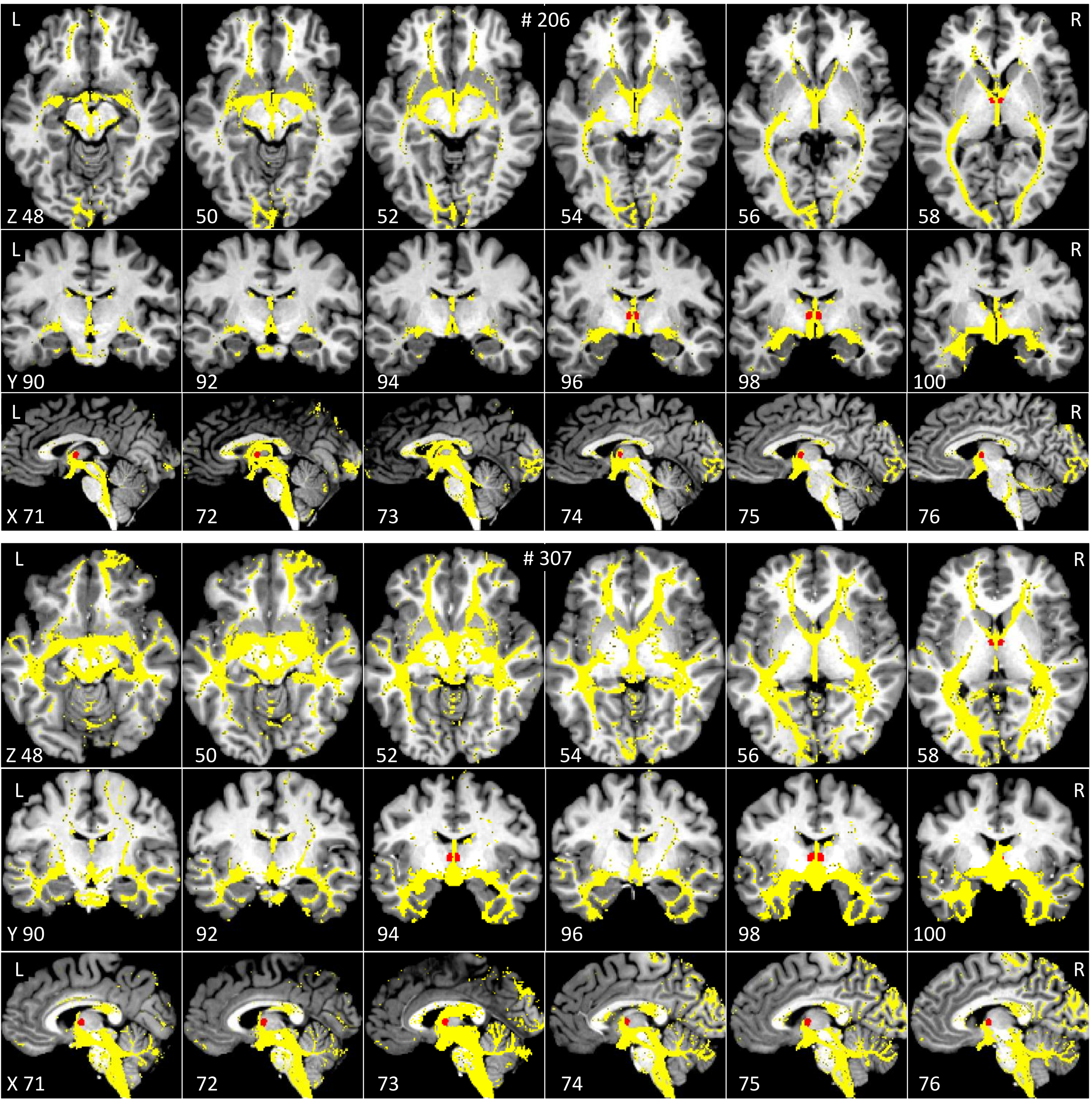

Suppl. Figure 3: Comparison of AM tracts of subject # 100206 and # 100307 in a) axial, b) coronal axial, c) sagittal views with the assignment of the slice positions in native diffusion space (T1 aligned to non diffusion image).

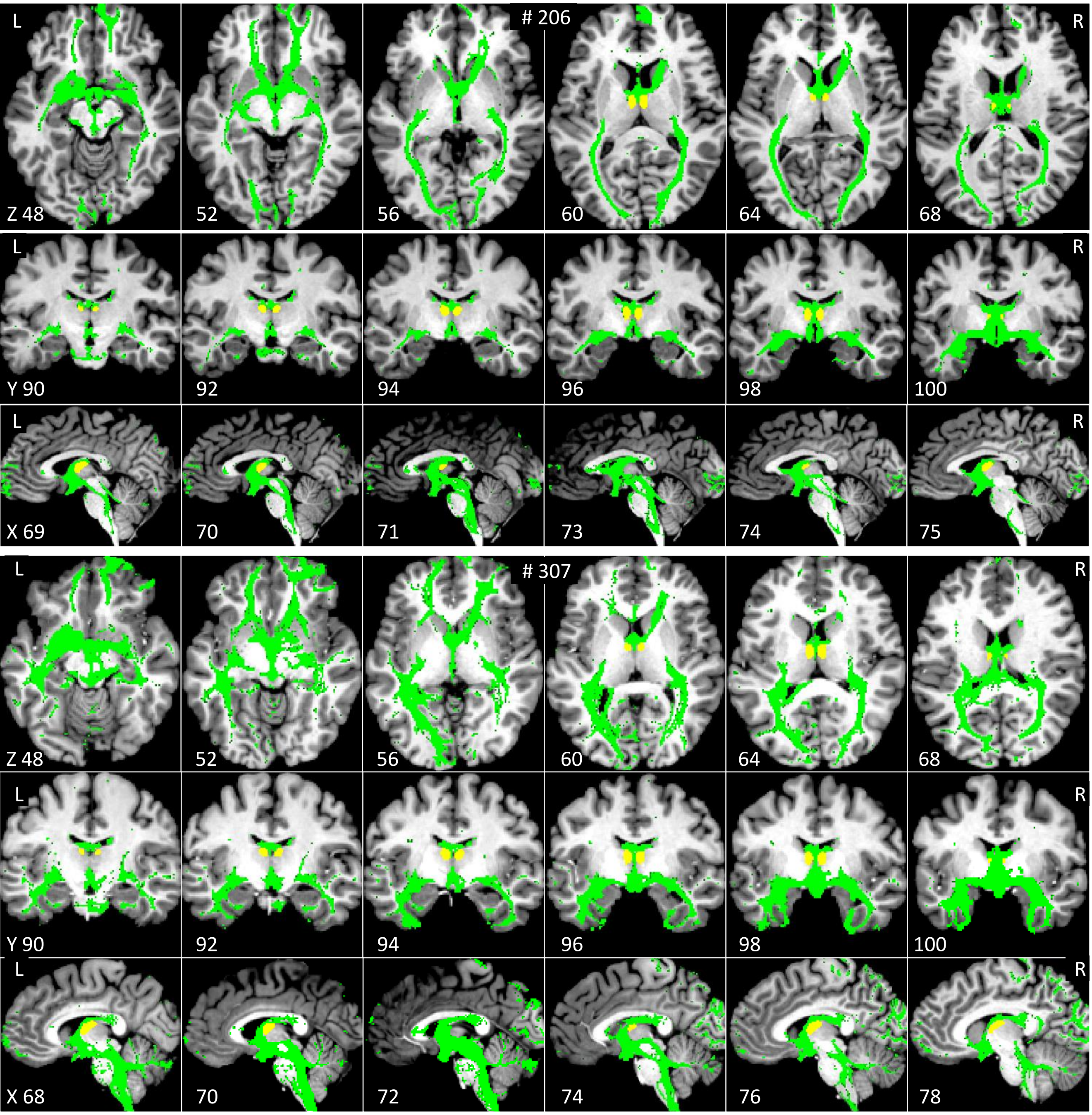

Suppl. Figure 4: Comparison of AV tracts of subject # 100206 and # 100307 in a) axial, b) coronal axial, c) sagittal views with the assignment of the slice positions in native diffusion space (T1 aligned to non diffusion image).

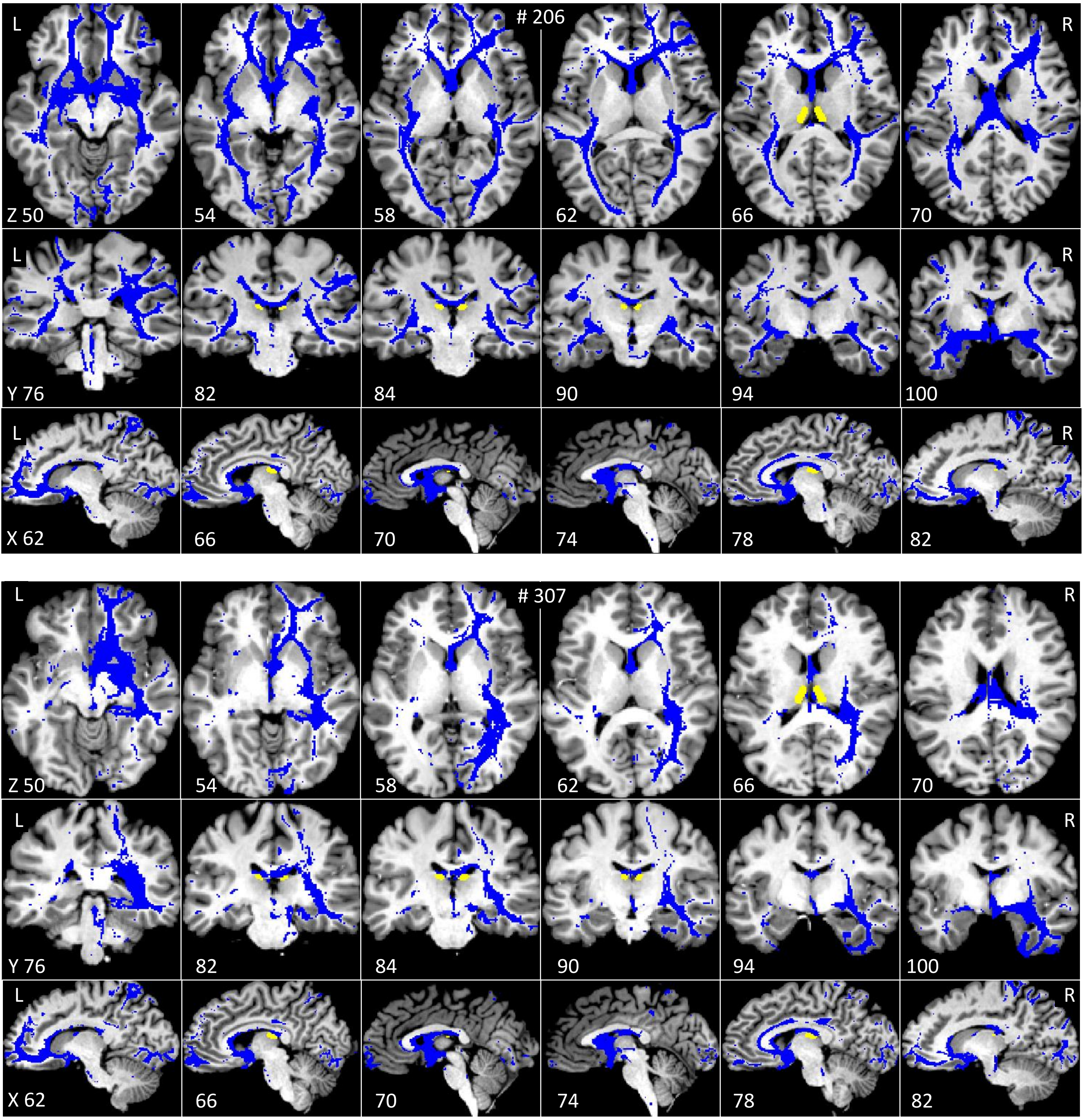

Suppl. Figure 5: Comparison of LD tracts subject # 100206 and # 100307 in a) axial, b) coronal axial, c) sagittal views with the assignment of the slice positions in native diffusion space (T1 aligned to non diffusion image).

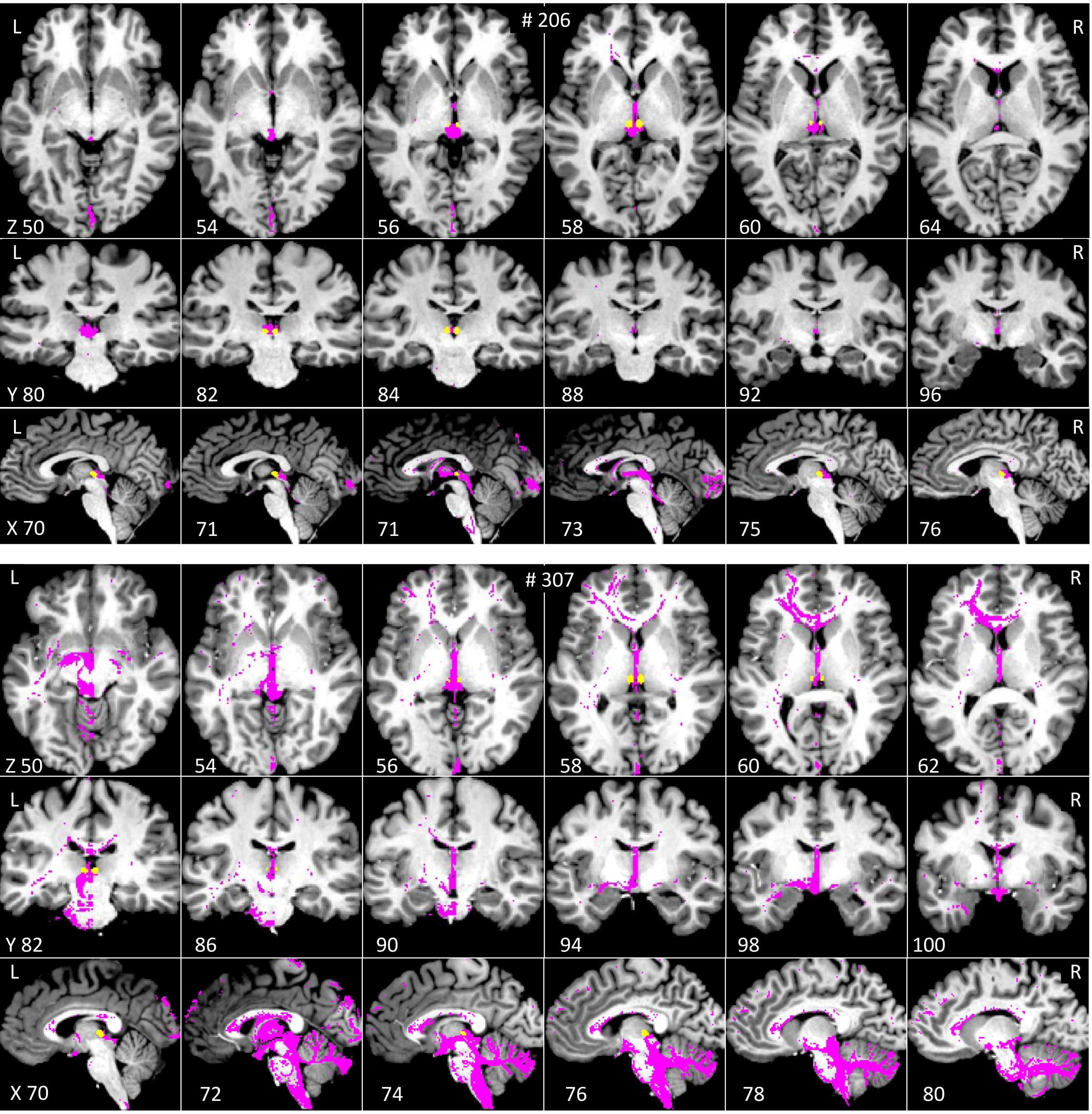

Suppl. Figure 6: Comparison of Hb tracts of subject # 100206 and # 100307 in a) axial, b) coronal axial, c) sagittal views with the assignment of the slice positions in native diffusion space (T1 aligned to non diffusion image).

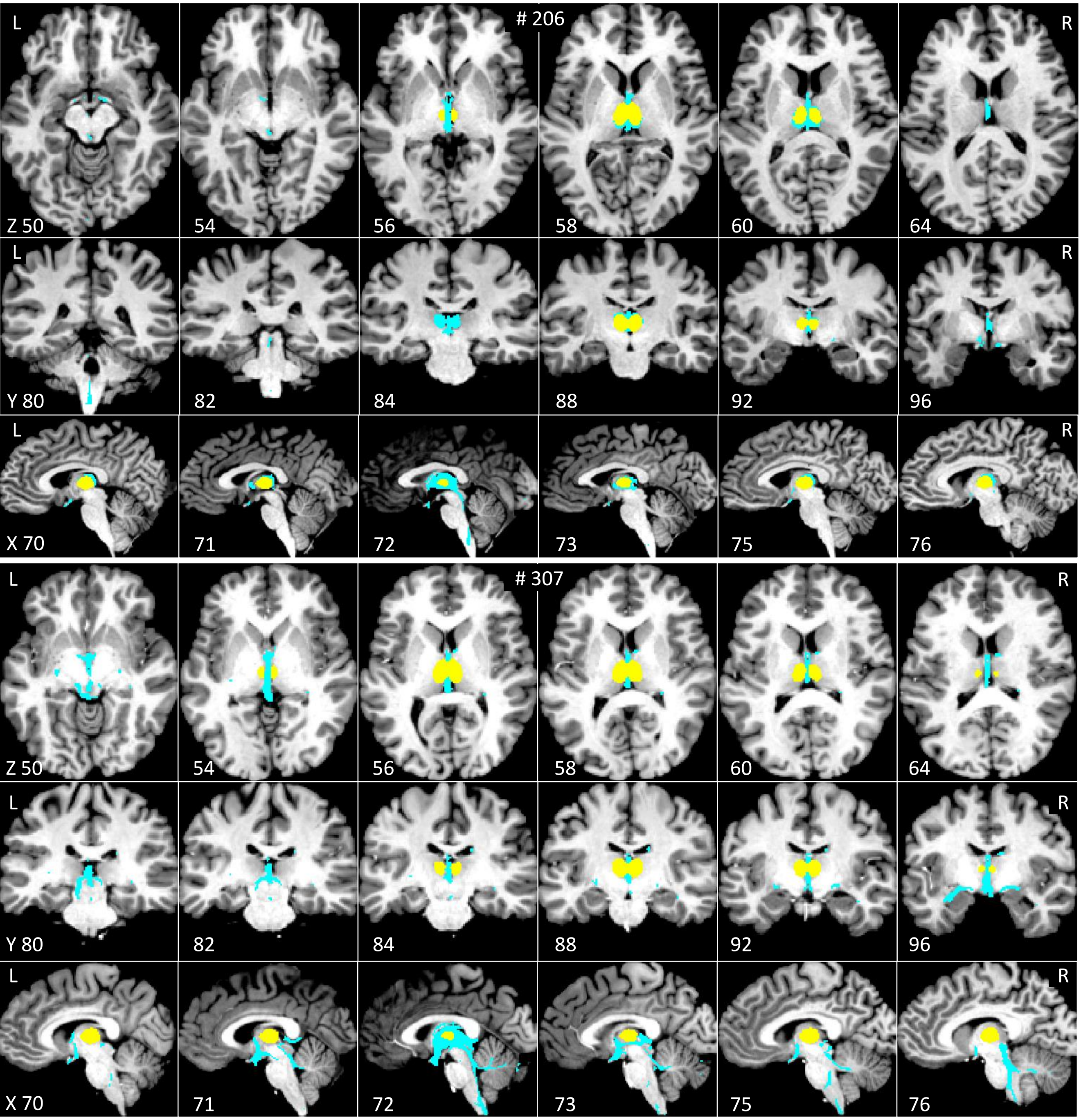

Suppl. Figure 7: Comparison of MD tracts of subject # 100206 and # 100307 in a) axial, b) coronal axial, c) sagittal views with the assignment of the slice positions in native diffusion space (T1 aligned to non diffusion image)..
